# A comprehensive mapping of the structure and gene organisation in the sheep MHC class I region

**DOI:** 10.1186/s12864-015-1992-4

**Published:** 2015-10-19

**Authors:** N. Siva Subramaniam, EF Morgan, JD Wetherall, MJ Stear, DM Groth

**Affiliations:** School of Biomedical Sciences, CHIRI Biosciences Research Precinct, Faculty of Health Sciences, Curtin University, GPO Box U1987, Perth, 6845 WA Australia; Department of Animal Production and Public Health, Faculty of Veterinary Medicine, University of Glasgow, Bearsden Road, Glasgow, G61 1QH UK; Institute of Biodiversity, Animal Health and Comparative Medicine, Garscube Estate, University of Glasgow, Bearsden Road, Glasgow, G61 1QH UK

**Keywords:** Major histocompatibility complex (MHC), MHC class I, Sheep, Mapping

## Abstract

**Background:**

The major histocompatibility complex (MHC) is a chromosomal region that regulates immune responsiveness in vertebrates. This region is one of the most important for disease resistance because it has been associated with resistance or susceptibility to a wide variety of diseases and because the MHC often accounts for more of the variance than other loci. Selective breeding for disease resistance is becoming increasingly common in livestock industries, and it is important to determine how this will influence MHC polymorphism and resistance to diseases that are not targeted for selection. However, in sheep the order and sequence of the protein coding genes is controversial. Yet this information is needed to determine precisely how the MHC influences resistance and susceptibility to disease.

**Methods:**

CHORI bacterial artificial chromosomes (BACs) known to contain sequences from the sheep MHC class I region were sub-cloned, and the clones partially sequenced. The resulting sequences were analysed and re-assembled to identify gene content and organisation within each BAC. The low resolution MHC class I physical map was then compared to the cattle reference genome, the Chinese Merino sheep MHC map published by Gao, et al. (2010) and the recently available sheep reference genome.

**Results:**

Immune related class I genes are clustered into 3 blocks; beta, kappa and a novel block not previously identified in other organisms. The revised map is more similar to Bovidae maps than the previous sheep maps and also includes several genes previously not annotated in the Chinese Merino BAC assembly and others not currently annotated in the sheep reference chromosome 20. In particular, the organisation of nonclassical MHC class I genes is similar to that present in the cattle MHC. Sequence analysis and prediction of amino acid sequences of MHC class I classical and nonclassical genes was performed and it was observed that the map contained one classical and eight nonclassical genes together with three possible pseudogenes.

**Conclusions:**

The comprehensive physical map of the sheep MHC class I region enhances our understanding of the genetic architecture of the class I MHC region in sheep and will facilitate future studies of MHC function.

**Electronic supplementary material:**

The online version of this article (doi:10.1186/s12864-015-1992-4) contains supplementary material, which is available to authorized users.

## Background

The major histocompatibility complex (MHC) is a highly polymorphic gene-dense region, spanning an area of approximately 4 Mbp in the human genome [1, 2]. Since its first discovery in mice [3], the MHC has been intensely studied in many species due to its association with immune related functions [2, 4–8]. In domestic animals, this has included the evolutionary relationship of the MHC in different species, the genetic diversity of animals subjected to domestication, its role in the immune response to parasites, its association with infectious and parasitic diseases and the development of vaccines [9–15].

In sheep, characterisation of the MHC has been based predominantly on analysis of orthologous loci from the respective human and cattle MHCs. Early studies have assumed that the basic structure of the sheep MHC was similar to that of other mammals, consisting of the telomeric class I, central class III and centromeric class II. A later study of MHC structure in Chinese Merino sheep reported that, like the cattle MHC [16], the sheep class II region is sub-divided into two distinct IIa and IIb regions and is most likely derived from a common ancestral partial chromosomal inversion [16, 17]. In recent years, low resolution physical maps of sheep MHC class II and III regions have been constructed using a combination of sub-cloning and partial sequencing of Bacterial Artificial Chromosome (BAC) clones known to contain MHC sequences [18, 19]. In addition, a panel of single nucleotide polymorphisms (SNPs) spanning the sheep MHC class II and III regions have also been developed [18, 19]. These have provided a framework for the identification and analysis of haplotypes.

However, a relative paucity in the knowledge regarding the sheep MHC still exists, in particular the class I region in terms of its gene content, structural organisation and genetic variation. Research into the MHC of sheep is currently limited in comparison with other domestic animals, especially cattle and swine [4, 8, 20–23]. For instance, there is a lack of understanding of its haplotype structure. Although dinucleotide microsatellite loci such as OHCCI [24] have been widely used in association studies in sheep [25–29] there is still a lack of understanding and characterisation of haplotypes in this important genetic region. The better understood human MHC map indicates that the class I region is rich in pseudogenes, duplicated genes and genes showing copy number variation [1].

Recently, a physical map of sheep MHC derived from a Chinese Merino sheep has been published by Gao and colleagues [30]. Annotation of this Chinese Merino physical map led to the identification of a total of 177 genes, among which 145 of the genes were apparently not previously identified in sheep and 10 described as unique to sheep [30]. From their study, 65 genes were reported in the MHC class I region. Twenty two predicted genes had either high sequence similarity to other known gene sequences, or contained a predicted open reading frame (ORF) having low sequence similarity with sequences from other species [30]. Three novel sheep-specific genes were also reported with no apparent sequence homology to any known mammalian sequences [30].

The objective of this study was to reanalyse existing and new information regarding the sheep class I region and produce a comprehensive and updated version of the sheep MHC class I map. This was achieved through the mapping of genes sequenced from CHORI BACs and comparing the result with the cattle reference genome [31], the previously published Chinese Merino sheep map [30] and the very recently available sheep reference genome [32]. In this study, we sub-cloned CHORI BACs known to contain class I sequences and re-assembled the sequences in order to annotate genes present within the sheep MHC class I region. In addition, we reanalysed, re-assembled and re-annotated the Chinese Merino BACs published by Gao and colleagues [30]. Annotation of the CHORI BAC and Chinese Merino BAC sequences was then used to generate a revised contig map. Comparison with the recent annotation of chromosome 20 from the sheep genome reference sequence [32] was then used to further inform this map.

## Results

### Re-analysis of Chinese Merino MHC contig map

**Fig. 1:**
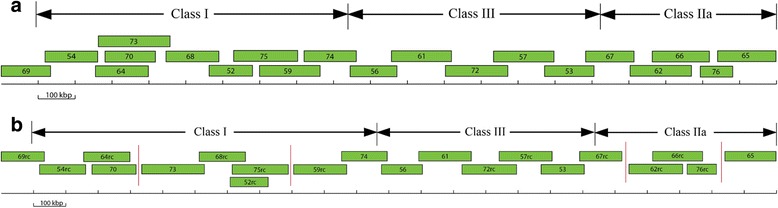
Comparison of the old and new tiling path of Chinese Merino BACs. **a** Original tiling path of the Chinese Merino MHC map (Gao et al. 2010). **b** New tiling path of Chinese Merino BAC sequences proposed in this study. A *vertical red line* between BAC contigs indicates a gap region

Initial analysis with Geneious 5.5 software produced 5 distinct contigs instead of the single contig reported by Gao et al. (2010). The Geneious assembly also revealed that some of the BAC sequences were not in the correct (5′–3′) orientation (Table 1). Four reads were not incorporated into the assembly; GenBank: FJ985852, GenBank: FJ985862, GenBank: FJ985865 and GenBank: FJ985867. Comparison with the published Chinese Merino map (Fig. 1) in the 5′ to 3′ direction: Contig 2 assembles four reads - GenBank: FJ985869, GenBank: FJ985854, GenBank: FJ985864 and GenBank: FJ985870. However, an overlap between GenBank: FJ985873 and either GenBank: FJ985864 or GenBank: FJ985870 was not detected. Contig 3 assembles 3 reads - GenBank: FJ985873, GenBank: FJ985868 and GenBank: FJ985875. GenBank: FJ985852 was not included in the assembly with GenBank: FJ985868 and GenBank: FJ985875. No overlap between GenBank: FJ985875 and GenBank: FJ985859 was identified. Contig 4 assembles GenBank: FJ985874 and GenBank: FJ985859. Analysis using Geneious did not identify any overlap between GenBank: FJ985874 and GenBank: FJ985856. Contig 1 assembles GenBank: FJ985856, GenBank: FJ985861, GenBank: FJ985872, GenBank: FJ985857 and GenBank: FJ985853. No overlap between GenBank: FJ985853 and GenBank: FJ985867, GenBank: FJ985867 and GenBank: FJ985862, GenBank: FJ985862 and GenBank: FJ985866 was identified. Contig 5 assembles GenBank: FJ985876 and GenBank: FJ985866. No overlap between GenBank: FJ985876 and GenBank: FJ985865 was detected by the Geneious algorithm. Contigs 2, 3 and 4 are in the opposite orientation when compared to the published Chinese Merino map [30] (Fig. 1a). Further analyses using the NCBI BLAST option to align two sequences and the CHAOS/DIALIGN software detected an overlapping region between the Chinese Merino BACs (GenBank: FJ985874 with GenBank: FJ985856) that was not reported by Geneious. In addition, analyses of BACs GenBank: FJ985852, GenBank: FJ985862, and GenBank: FJ985867 that had been omitted in the initial assembly using the Geneious analysis showed overlapping sequence alignment with other Chinese Merino BACs; GenBank: FJ985852 overlaps with GenBank: FJ985868 and GenBank: FJ9858675, GenBank: FJ985862 overlaps with GenBank: FJ985866, and GenBank: FJ985867 overlaps with GenBank: FJ985853. Figures S1 and S2 in Additional file 1 provides the results of these analyses as a series of pairwise dotplots for the BACs along with an interpretive comment. The regions of overlaps between the BAC sequences are detailed in Table 2.

**Table 1:** Geneious assembly of 20 BAC clones published by Gao et al. (2010)

Assembled reads	Length (bp)	Reads unused	Length (bp)
Contig 1: 5 Reads	629701	FJ985852	118738
FJ985856 (f)	127050	FJ985862	167309
FJ985861 (f)	162317	FJ985865	159959
FJ985872 (r)	169910	FJ985867	133881
FJ985857 (r)	165531		
FJ985853 (f)	134434		
Contig 2: 4 Reads	430168		
FJ985870 (r)	138311		
FJ985864 (f)	142360		
FJ985854 (f)	145292		
FJ985869 (f)	134643		
Contig 3: 3 Reads	460095		
FJ985875 (f)	173955		
FJ985868 (f)	140835		
FJ985873 (r)	196844		
Contig 4: 2 Reads	283944		
FJ985874 (r)	141902		
FJ985859 (f)	160643		
Contig 5: 2 Reads	214322		
FJ985876 (f)	88495		
FJ985866 (f)	155022		

(f) indicates assembly of sequence in the forward (5′- > 3′) direction. (r) indicates assembly of the reverse complement sequence (3′- > 5′)

**Table 2:** Overlapping regions of Chinese Merino BACs in a telomeric to centromeric (5′ to 3′) orientation

BAC IDs	3′ Loc	5′ Loc
69rc x 54rc	124243–134643	1–10399
54rc x 64rc	140977–145292	765–5061^a^
64rc x 70	-	-
64rc x 73	-	-
70 x 73	26368–142360	1–116126
73 x 68rc	179850–196844	1–16758
68rc x 52rc	94483–140835	1–46198
68rc x 75rc	105191–140835	1–35324
52rc x 75rc	10710–118738	1–108231
75rc x 59rc	-	-
59rc x 74	142004–160643	1–18619
74 x 56	121429–141902	1–20418
56 x 61	115294–127050	1–11759
61 x 72rc	134589–162317	1–27705
72rc x 57rc	114820–169910	1–55107
57rc x 53	130181–16553	1–35410
53 x 67rc	118698–134434	1–15709
67rc x 62rc	-	-
62rc x 66rc	74418–167309	1–94777
66rc x 76rc	125570–155022	1–29542
76rc x 65rc	-	-

Precede BAC ID numerals with ‘FJ9858’ to determine NCBI GenBank accession. Appended ‘rc’ indicates the BAC was reverse complemented before alignment. ‘-‘ indicates there was no overlap identified. ^a^First 765 bp of GenBank: FJ985864rc do not overlap with GenBank: FJ985854rc, this is most likely due to contig misassembly in this region

A pictorial view of our revised tiling map of the Chinese Merino BAC sequences is shown in Fig. 1b. Based on this analysis, there are gaps between BACs GenBank: FJ985870 and GenBank: FJ985873, GenBank: FJ985875 and GenBank: FJ985859, GenBank: FJ985867 and GenBank: FJ985862, and GenBank: FJ985876 and GenBank: FJ985865. Blast analysis of the first 764 nucleotides of the reverse complemented GenBank: FJ985864 indicated that nucleotides 1–359 aligned perfectly, beginning 26,374 nucleotides downstream in the same BAC, and nucleotides 403–764 aligned perfectly, beginning 35,659 nucleotides downstream also in the same BAC, with a gap of approximately 8900 bp between the two alignments (data not shown). The sequence between bp 360 and 403 is a string of undefined nucleotides, hence no alignment was obtained in this region. We conclude that these 764 nucleotides have not been assembled correctly. Subsequent dot plot alignment of the Chinese Merino BACs with the MHC Class I region of the reference sheep chromosome 20 is presented in the "Comparative analysis of CHORI BAC contigs map with MHC Class I maps" Results section below.

### Analysis of sheep MHC class I gene content

**Fig. 2:**
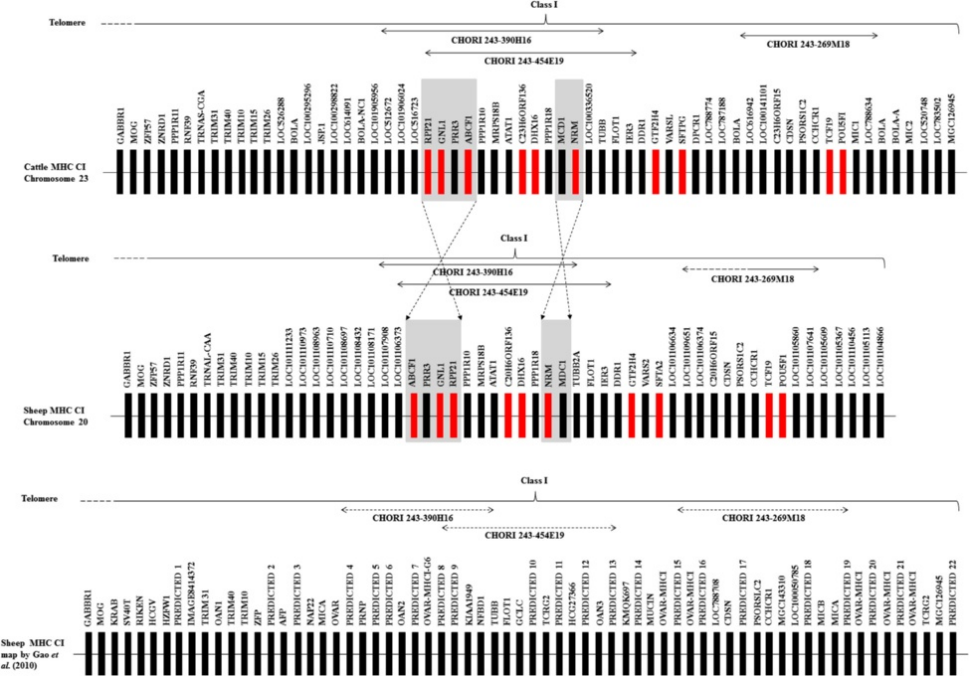
Comparison of cattle (NCBI), sheep (NCBI) and Chinese Merino MHC Class I maps. Loci highlighted in *red* indicate additional loci identified through sequencing of CHORI BAC clones. Additional file 3: Table S1 and S2 provides the detailing of gene symbol, gene name and gene description in the class I region of the sheep [GenBank: NC_019477] and cattle [GenBank: AC_000180] reference genomes at NCBI, respectively

**Fig. 3:**
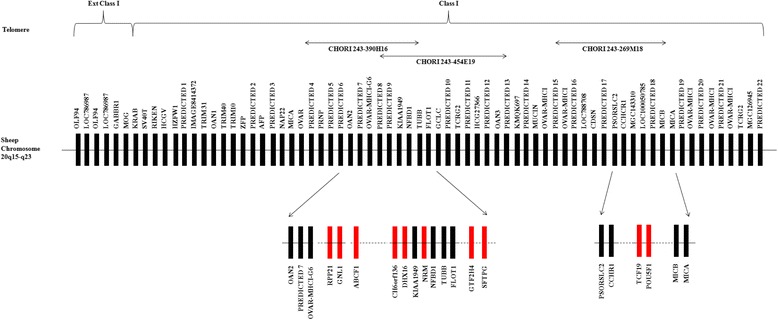
Loci identified through CHORI BAC sub-cloning and their relative position within the Chinese Merino map. Identity of these loci was confirmed by sequence homology using NCBI BLAST. Loci highlighted in *red* indicate loci previously not present on the map proposed by Gao et al. (2010)

Initial analysis of CHORI BAC sub-clone sequences identified 10 additional loci in the sheep MHC class I region that were not recognised in the Chinese Merino MHC map [30] but were found in both the cattle and sheep reference maps. Comparison of MHC class I gene content between the cattle reference map, sheep reference map and the published Chinese Merino map [30] is given in Fig. 2. The additional loci identified through CHORI BAC sub-clones include *ribonuclease P 21-like isoform 2* (*RPP21*), *guanine nucleotide-binding protein-like 1 (GNL1)*, *ATP-binding cassette sub-family F member 1 (ABCF1)*, *chromosome 6 open reading frame 136 ortholog (C20H6orf136)*, *DEAH (Asp-Glu-Ala-His) box polypeptide 16 (DHX16)*, *nuclear envelope membrane protein* or *nurim (NRM)*, *general transcription factor II H subunit 4 (GTF2H4)*, *surfactant associated protein G (SFTPG)*, *transcription factor 19 (TCF19)* and *POU class 5 homeobox 1 (POU5F1)*. The relative locations of these additional loci were deduced using BLAST analysis. Figure 3 shows the positions of the additional loci identified through sequencing of these CHORI BAC subclones that had not been identified in the previously published Chinese Merino MHC map [30].

Our re-analysis of the MHC CI Chinese Merino map indicates that the majority of genes identified appear to have the same gene arrangement with that previously observed in cattle, along with a high level of nucleotide similarity to their cattle orthologues. The name and location of genes identified in the Chinese Merino BAC sequences are all tabulated in Table 3. Identical gene predictions between adjacent BAC sequences corroborates with overlapping regions identified through dotplot and pairwise sequence alignment analysis. Likewise, non-overlapping regions show no similarity in gene content.

**Table 3:**
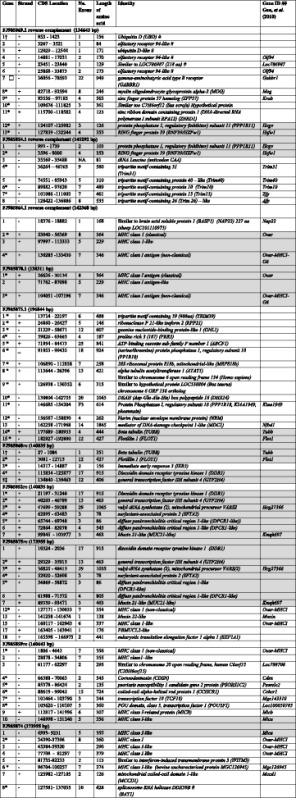
Summary of gene content on each of the Chinese Merino BAC sequences

†First exon not in BAC. ‡Last exon not in BAC. *Alignment to known homolog(s) shows high percent identity with no evidence of wrong or missing exons. # not included in Fig. 4. Shaded regions indicate genes identified in overlapping regions between clones. ## Identified from Additional file 3: Table S1 (Gao et al. 2010). Gene IDs not matching our gene symbols were surmised based on gene description and/or alternate gene symbols provided in the NCBI Gene database along with relative gene position compared to our analysis

The majority of gene predictions in the overlapping regions of the Chinese Merino BACs were identical, however there were a few exceptions. In the overlap region between GenBank: FJ985852rc and GenBank: FJ985875rc, there were differences observed in the *VARS2* and *DPCR1-like* predictions. Exon 27 of the *VARS2* gene prediction is longer in GenBank: FJ985852rc than in GenBank: FJ985875rc due to a single bp deletion in the exon region in GenBank: FJ985875rc that results in a change in the sequence reading frame and hence a premature stop to the exon. Alignment with reference sequences indicates that the *VARS2* gene prediction from GenBank: FJ985852rc is correct (data not shown). Exon 2 of the *DPCR1-like* gene prediction is shorter in GenBank: FJ985852rc than in GenBank: FJ985875rc due to a significant insertion of ~370 bp in GenBank: FJ985875rc compared to GenBank: FJ985852rc. Alignment with reference sequences indicates that the GenBank: FJ985852rc prediction is more similar and hence more likely to be correct. In the overlapping region between GenBank: FJ985859rc and GenBank: FJ985874, there is a single amino acid length difference noted in the GenBank: FJ985874 MHC Class I-like predicted peptide compared to the GenBank: FJ985859rc peptide due to a 3 bp deletion in the region of the first exon. An alignment with reference sequences failed to indicate which sequence is correct since the difference occurs in an area of low complexity repeats within the signal peptide.

A comparison between genes reported in the Chinese Merino map by Gao et al. [30] and our analysis showed that twenty-nine of the sixty-eight genes identified in this study were not previously annotated. The ten genes identified through sub-cloning and sequencing of the CHORI BAC sequences were annotated in the re-analysis of Chinese Merino BAC sequences. Table 3 includes a list of gene identifications reported by Gao et al. [30] for the thirty-nine genes identified in both studies.

### Sequence comparison of Chinese Merino BACs with MHC class I region of reference sheep chromosome 20

Chinese Merino BACs covering the MHC Class I region were aligned via dotplot analysis with a portion of the sheep chromosome 20 from the MHC class I region downloaded from NCBI [GenBank: NC_019477, bp 27564303–28934000]. The results of this Dotplot analysis are presented in Additional file 2: Figure S3.

BACs GenBank: FJ985854, GenBank: FJ985868 and GenBank: FJ985852 align well with the sheep reference genome sequence in this region, there are no obvious large indels and no significant interspersed repetitive regions. A large indel is observed in the alignment between GenBank: FJ985869 (reverse complemented sequence) and GenBank: NC_019477 due to the presence of ~25,000 bp in the sheep reference sequence not found in the GenBank: FJ985869 BAC. The insertion in the sheep genome is approximately from position 280689920 to 28093580 in GenBank: NC_019477. Within this region there are two annotated genes - described as an *ubiquitin D-like* gene and *C19orf12* homolog – that were not located in the GenBank: FJ985869 BAC. Smaller indels and gaps in the alignment diagonal appear to correspond to runs of undefined nucleotides within the sheep reference sequence. Shorter runs of undefined nucleotides are also present in the BAC sequence.

BACs GenBank: FJ985864 and GenBank: FJ985870 overlap over the majority of their sequence, and this is evident when comparing their alignments to GenBank: NC_019477. There are several indels, and the dotplot shows parallel lines indicating large interspersed repeating regions. These BACs contain several MHC class I histocompatibility antigen-like loci, which share enough sequence similarity to create an interspersed pattern of diagonals. Within the corresponding region on GenBank: NC_019477, larger gaps in the alignment are evidence of possible mis-assembly in either the BAC or sheep reference chromosome 20, or could also represent breed specific differences in the region. The alignment of FJ985864 with GenBank: NC_019477 also reveals that the first 764 nucleotides align in two different segments 5′ to the main alignment diagonal, with a gap of approximately 9000 bp between, confirming the observation from BLAST analysis of this region that these first 764 nucleotides are mis-assembled (refer to "Re-analysis of Chinese Merino MHC contig map" in Results section).

**Fig. 4:**
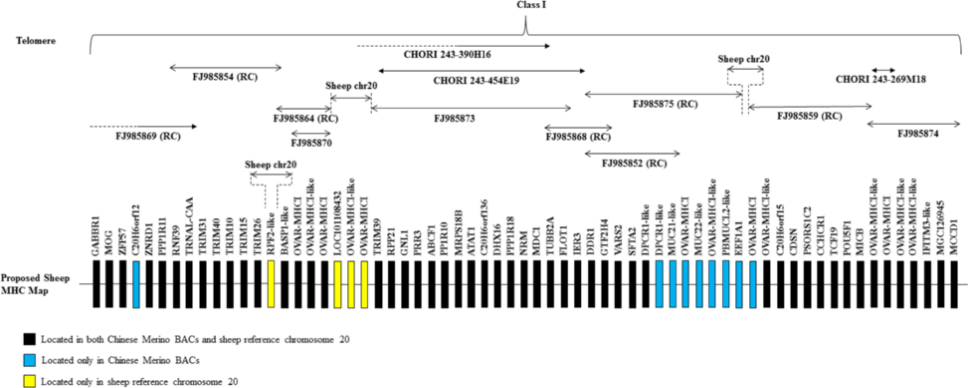
The new sheep MHC CI map proposed based on findings in this study. Position of each locus is based on the comparative analysis between CHORI BACs, Chinese Merino BACs, cattle reference sequence for chromosome 23 [GenBank: AC_000180] and sheep reference sequence for chromosome 20 [GenBank: NC_019477]

The alignment of GenBank: FJ985873 with GenBank: NC_019477 reveals a segmental inversion covering an area of approximately 75,000 bp. Within this region are four genes that have been predicted in reverse orientation within the two sequences – *ABCF1*, *PRR3*, *GNL1* and *RPP21* (refer Figs. 2 and 4). The inverted sequence may be a result of mis-assembly in this region in either the sheep reference chromosome 20 or the Chinese Merino BAC; alternatively, it may represent a genuine breed specific difference. In cattle and in the Chinese Merino BAC sequence, the genes are annotated in the order *RPP21*, *GNL1*, *PRR3*, *ABCF1*. This order was chosen for our proposed map (Fig. 4) as it is consistent with the order annotated (telomeric to centromeric) in other species including *Bos taurus*, *Sus scrofa* and *Mus musculus*.

The dotplot analysis confirmed a gap between Chinese Merino BAC sequences GenBank: FJ985870 and GenBank: FJ985873 of 49,078 bp, lying in the region from 27555826–27604904 in GenBank: NC_019477. Annotated within this region in the sheep reference genome are two MHC class I histocompatibility antigen–like genes (hereafter referred to as MHC class I-like genes) and an envelope glycoprotein-like gene.

The reverse complement of GenBank: FJ985875 aligns well with sheep reference chromosome 20 for ~107,000 bp. There is no significant alignment from ~107,000 bp to the end of the sequence - ~67,000 bp. GenBank: NC_019477 has a 5000 bp run of undefined nucleotides spanning the region from ~107,000 to 112,000 bp in GenBank: FJ985875 and 27147579 to 27152580 in GenBank: NC_019477. The remaining sequence in GenBank: FJ985875 represents inserted sequence not currently present in the sheep reference genome. This is confirmed in the dotplot alignment with the reverse complement of GenBank: FJ985859, which spans a region in GenBank: NC_019477 from bp 27007323 to 27147579. Five genes are annotated in the region from bp 112,000 to the end of GenBank: FJ985875 (bp 173,955) including two *MHC class I-like* genes, two *mucin-like* genes and *eukaryotic translation elongation factor 1 alpha 1 (EEF1A1). MHC class I* and *mucin-like* genes would be expected in the MHC class I region, however *EEF1A1* has not been annotated in the MHC Class I region in genomes from other closely related species, including cow, pig, mouse and human.

The first 23,000 nucleotides of the reverse complement of GenBank: FJ985859 do not align with GenBank: NC_019477. 5000 bp may be accounted for by undefined nucleotides in GenBank: NC_019477, however the remainder represents an inserted region of ~18,000 bp not currently present in the sheep reference genome. Within this region in GenBank: FJ985859 one MHC class I histocompatibility antigen gene has been predicted. Several interspersed repeat regions are indicated in the alignment bp of the reverse complement of GenBank: FJ985859 in a ~9000 bp span between bp 144,000 and 153,000. Within this region an MHC class I-like gene has been predicted.

Comparison of the dotplot alignments between the reverse complement of GenBank: FJ985859 and GenBank: NC_019477 and between GenBank: FJ985874 and GenBank: NC_019477 indicates an overlap between GenBank: FJ985859 and GenBank: FJ985874 of 18,663 bp. The dotplot alignment of GenBank: FJ985874 with GenBank: NC_019477 shows a mostly unbroken alignment diagonal. There are two noticeable indels of approximately 1500 bp that are the result of undefined nucleotides in the sheep reference genome sequence. A number of interspersed repeats are indicated in the first ~80,000 bp of GenBank: FJ985874, within this region there are four predicted MHC class I – like genes.

Analysis of the dotplot alignments confirms the overlaps of the Chinese Merino BAC sequences as indicated in Fig. 1b. These are summarised in Table 4. Dotplot analysis provides no evidence of overlap between GenBank: FJ985870 and GenBank: FJ985873 or between GenBank: FJ985875 and GenBank: FJ985859. We were unable to estimate the size of the gap between GenBank: FJ985875 and GenBank: FJ985859.

**Table 4:** Overlaps of BACs published by Gao et al. (2010) with sheep reference genome sequence GenBank: NC_019477

Overlap	Length (bp)
FJ985869rc with FJ985854rc	9965
FJ985854rc with FJ985864rc	4069
FJ985864rc with FJ985870	130,103
FJ985873 with FJ985868rc	16690
FJ985868rc with FJ985852rc	46429
FJ985852rc with FJ985875rc	108200
FJ985859rc with FJ985874	18663

### Comparative analysis of CHORI BAC contigs map with MHC Class I maps

Comparative analysis of the assembled, sub-cloned CHORI BAC sequences revealed the coverage of each BAC and its position with respect to the cattle reference sequence for chromosome 23 [GenBank: AC_000180], Chinese Merino BAC sequences [30] and the sheep reference sequence for chromosome 20 [GenBank: NC_019477] (Table 5).

**Table 5:** BLAST analysis of CHORI BAC end sequences available in GenBank. The sequences were aligned with cattle and sheep reference sequences from chromosome 20 and 23, respectively, and with the Chinese Merino BAC sequences

BAC ID	BAC end sequence GenBank ID	Location within GenBank: AC_000180 (Cattle)		Location within GenBank: NC_019477 (Sheep)		Location within Chinese Merino BACs		
CH243-	GenBank: DU202647.1	28318396–28318799	Between LOC512672 and LOC101905956	27582535–27582944	Between LOC101107908 and LOC101108171	No significant similarity found		
390H16								
	GenBank: DU201205.1	28104186–28104742	Tubulin, beta 2B (TUBB)	27366724–27367274	Tubulin, beta 2A (TUBB2A)	FJ985873	178105–178656	Beta tubulin (TUBB)
CH243-	GenBank: DU252291.1	28268052–28268910	Tripartite motif-containing 39 (TRIM39)	27538705–27539533	LOC101106373	FJ985873	16302–17130	Tripartite motif-containing 39 (TRIM 39)
454E19								
	GenBank: DU262410.1	28071261–28072253	Between discoidin domain receptor family, member 1 (DDR1) and immediate early response 3 (IER3)	27332144–27332884	Between discoidin domain receptor family, member 1 (DDR1) and immediate early response 3 (IER3)	FJ985868RC	32632–33370	Between discoidin domain receptor family, member 1 (DDR1) and immediate early response 3 (IER3)
CH243-	GenBank: DU418632.1	27849059–27849865	Between LOC616942 and BOLA (both MHC class I-like)	26997887–26998048	Between LOC101105609 and LOC101107641 (both MHC class I-like)	FJ985859RC	18419–19215	Between MHC class I and MHC class I-like
269 M18								
	GenBank: DU420388.1	27697572–27698209	Between LOC788634 and BOLA (both MHC class I-like)	26982178–26982955	Within MHC class1-like	FJ985859RC	157679–158508	Between MHC class I-like genes
						FJ985874	15655–16484	Between MHC class I-like genes

The telomeric end sequence of CHORI 243-390H16 [GenBank: DU202647] aligns between two *MHC class I-like* loci in both the cattle and sheep reference sequences, but shows no significant similarity to any of the Chinese Merino BAC sequences. It appears that this CHORI BAC end sequence lies within a gap region between Chinese Merino BAC GenBank: FJ985870 and GenBank: FJ985873. The centromeric end sequence of CHORI 243–390H16 [GenBank: DU201205] aligns within the predicted *TUBB* locus in both cattle and sheep reference sequences and in Chinese Merino BAC GenBank: FJ985873.

The CHORI 243–454E19 BAC appears to span a region beginning between loci *DDR1* and *IER3,* and ending within a *TRIM 39-like* locus in the cattle and sheep reference sequences and in the Chinese Merino BACs. CHORI 243–390H16 overlaps with CHORI 243–454E19 and was located in the middle of MHC class I region whereas, CHORI 243–269 M18 was located further away towards the centromeric end. Both the telomeric and centromeric ends of CHORI 243–269 M18 are located between 2 *MHC class I-like* genes with respect to the cattle and sheep reference sequences and in the Chinese Merino BACs. Using this information Fig. 4 shows a revised sheep MHC class I map based upon our reanalysis.

### Comparative analysis of MHC class I histocompatibility antigen genes

**Fig. 5:**
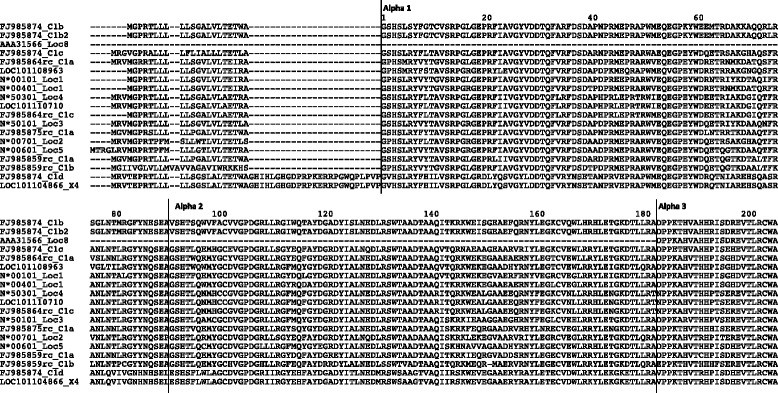
Shows an alignment of MHC class I amino acid sequences predicted from selected BAC sequences published by Gao et al., 2010 in comparison with selected class I loci from the sheep reference chromosome 20 (NCBI accession NC_019477.1) and six class I reference sequences from the IPD database (identified as N*xxxxx) plus an alternative transcript sequence AAA31566. Nomenclature for the predicted genes from BAC sequences derived as follows: NCBI accession number, plus ‘rc’ if the sequence was reverse complemented before analysis, plus the class I gene identifier within the BAC. Sheep reference sequences identified by gene symbol in the NCBI Gene database. IPD sequences identified according to IPD allele name in the IPD-MHC database (http://www.ebi.ac.uk/cgi-bin/ipd/mhc/view_nomenclature.cgi?ovar.n). AAA31566 identified as an allele of Locus 8 (_Loc8) by Ballinghall, et al. 2008. Vertical lines delineate functional domains. A vertical box surrounds a motif in the transmembrane region important in classification of class I sequences as classical or nonclassical in cattle and sheep

Of particular interest was the determination of the number and distribution of the class I histocompatibility antigen loci. In cattle, there is evidence for at least six classical and four nonclassical discrete MHC class I loci [33–35]. In sheep, evidence exists for at least six discrete class I loci, with at least two being classical [36, 37]. The number and expression levels of class I loci have been demonstrated to be haplotype specific in the Scottish Blackface sheep breed [36, 37], so loci identified in the Chinese Merino BACs are expected to differ from the sheep reference genome (Texel). Correct assembly of these highly repetitive gene sequences is bioinformatically a difficult task, compounded by variant numbers of loci in different haplotypes. MHC class I genes were predicted in the Chinese Merino BACs as detailed in the "Re-analysis of Chinese Merino sheep MHC map" Methods section and are included Table 3. Predicted MHC class I protein sequences from both the Chinese Merino BACs and the sheep reference sequence for chromosome 20 were aligned with selected reference sequences taken from the IPD-MHC database (http://www.ebi.ac.uk/cgi-bin/ipd/mhc/view_nomenclature.cgi?ovar.n). In addition, functional domain analysis was carried out for each sequence. Tables 6 and 7 list the class I genes predicted in the Chinese Merino BAC sequences and corresponding sheep reference chromosome 20 sequences, respectively, and include details on the location of signal peptides and functional domains identified. The MHC class I genes with identifiable transmembrane and cytoplasmic domains are denoted as classical (Ia) or nonclassical (Ib) according to sequence criteria previously used in cattle [33, 34, 38] and sheep [36, 37]: namely the presence of a VPI, IPI or VLIK motif in the transmembrane domain and/or a truncated cytoplasmic domain. A sequence alignment of the predicted class I proteins from the Chinese Merino BAC sequences in comparison with a selection of reference sequences representing different loci can be seen in (Additional file 4: Figure S4), A similar alignment with the predicted class I proteins from the sheep reference chromosome 20 sequences can be seen in (Additional file 4: Figure S5). Sequences considered to most likely represent functional class I genes are aligned in Fig. 5. The location of functional domains are also indicated for each of these alignments.

**Table 6:** MHC class 1-like genes predicted from genomic BAC sequences published by Gao et al. (2010)

Name	#	S	Location	#Ex	AA len	SP	♋1/♋2	♋3	TM	C-Term	Type	Homologue
FJ985864rc_C1a	1	+	53040–56369	8	364	1-25	26–203	207–299	310–331	336–363	Ia	NP_001124406
FJ985864rc_C1b	2	+	97997–113333	5^a^	229	No	27–93	95–186	194 215	No		NP_001124406
FJ985864rc_C1c	3	+	130285–133430	7	346	1–22	23–200	205–296	304–325	329–346^b^	Ib	NP_001124406
FJ985870_C1a	1	+	26626–30134	8	364	1–25	26–203	207–299	310–331	336–363	Ia	NP_001124406
FJ985870_C1b	2	+	71762–87098	5^a^	229	No	27-93	95-186	194 215	No		NP_001124406
FJ985870_C1c	3	+	104051–107196	7	346	1–22	23–200	205–296	304–325	329–346^b^	Ib	NP_001124406
FJ985875rc_C1a	4	-	127171–130033	7	354	1–25	26–203	207–299	307 –329	332–354	Ib	NP_001035644
FJ985875rc_C1b	5	-	160117–162943	6	337	1–25	26–187	189–233	242–262	267–279^b^	Ib	NP_001035644
FJ985859rc_C1a	6	-	1806–4642	7	356	1–25	26–203	207–299	308 327	332–356^b^	Ib	CAI43976
FJ985859rc_C1b	7	-	28878–34806	7	355	1-18	28-204	208-300	309 329	333–355	Ib	NP_001124406
FJ985859rc_C1c	8	-	148998-151240	5	356	1–25	52–229	233–325	333 350	No		AAZ74696
FJ985874C1a	8	-	6993-9231	5	355	1–24	51–228	232–324	332 349	No		AAZ74696
FJ985874C1b^c^	9	-	34484-37396	8	360	1–22	23–200	204–296	305 328	331–359	Ib	FJ985864rc_C1a
FJ985874C1b2^c^	9	-	34390-37396	7	383	1–22	23–200	204–296	305 328	331–359	Ib	CAI43976
FJ985874C1c	10	-	77708-81297	7	379	1–25	26–203	207-299	305 328	333–355^b^	Ib	NP_998933
FJ985874C1d	11	-	96704-100257	7	374	1–24	50–228	232–324	333–353	358–374^b^	Ib	NP_001070451

S: strand. #: Locus number in telomeric to centromeric direction. #Ex: number of exons identified. AA len: Length of predicted amino acid sequence. SP: signal peptide. α1: alpha 1 domain. α2: alpha 2 domain. α3: alpha 3 domain. TM: transmembrane domain. C-Term: C-terminus end. Homologue indicates sequence used as homologous protein sequence with FGENESH+

^a^Terminal exon not predicted. ^b^Weak match. ^c^Alternative transcript predictions

**Table 7:** Current annotated loci identified as class I histocompatibility antigen-like on the sheep Chromosome 20 Reference Oar_v3.1 primary assembly (NC_019477.1)

Gene Symbol	#	S	Location	#Ex	AA length	SP	*♋*1/*♋*2	*♋*3	TM	C-Term	Type	Protein Acc
OLA-I	1	-	27766021..27769569	7	359	1–21	22–200	204–296	305–326	331–358	Ia	NP_001295381
(LOC101108963)												
LOC101110710	2	-	27673886..27678484	7	349	1–24	25–203	208–299	307–328	333–349	Ib	NP_001295515
LOC101108171	3	-	27597572..27599984	4	408	1–21	110–284	288–379	No	No		XP_011956856
LOC101107908	4	+	27544800..27596662	X2: 7^a^	380	No	48–235	239–331	337–356	No		XP_011956356
				X3: 7	376	No	48–235	239–	337–	No		XP_011956357
				X4: 7	370	No	48–235	331	356	No		XP_011956358
				X1: 6	380	No	48–235	239–331	337–356	No		XP_011956355
								239–331	337–356			
LOC101106374	5	+	27139179..27141475	4	244	1–24	57–162	166–244	No	No		XP_011956854
LOC101105860	6	-	27024840..27032915	6	310	1–24	31–203	No	236–257	No		XP_011956400
LOC101107641	7		27016322..27018726	5	332	1–27	31–205	209–301	336–356	No		XP_011956401
LOC101105609	8		26988241..26996391	5	321	1–21	82–170	174–266	274–293	No		XP_011956852
LOC101105367	9		26963865..26967651	6	229	No	69–146	No	184–203	212–228	1b	XP_011956851
LOC101104866	10		26922240..26948265	X1: 8	430	1–25	79–253	257–349	400–417	264–345	1b	XP_011956402
				X4: 7	374	1.24	50–228	232–324	333–353	239–320	1b	XP_012018733

S: strand. #: Locus number in telomeric to centromeric direction. #Ex: number of CDS exons identified. AA len: Length of predicted amino acid sequence. SP: signal peptide. α1: alpha 1 domain. α2: alpha 2 domain. α3: alpha 3 domain. TM: transmembrane domain. C-Term: C-terminus end. Type: Ia – classical, Ib – nonclassical. Protein Acc: Accession of protein sequence in NCBI database

^a^Transcripts differ only at the C-terminal end

In summary, 11 putative MHC class I-like genes were located in the Chinese Merino BACs (refer to Table 6). Three were found in BACs FJ985864 and FJ985870; in each case the corresponding gene from these two BAC sequences was identical. This was not unexpected as these two BAC sequences overlap. BAC sequences FJ985875, FJ985859, FJ985874 contained two, three and four putative MHC class I genes respectively. The last gene in BAC FJ985859 sequence is identical to the first gene identified in BAC FJ985874 except for a 3 nucleotide indel, which most likely represents an allelic variation. Pairwise alignment of the two BACs indicates an overlap in the region containing the putative MHC class I gene. Predicted genes identified as FJ985874_C1b and FJ985874_C1b2 represent alternative transcripts of the same gene.

Similar analysis of the sheep reference chromosome 20 annotated class I sequences is shown in Table 7. Ten putative MHC class I loci have been identified. Based on the analysis of the protein sequences, one locus (LOC101108963) appeared to be a classical class I gene and there were three nonclassical genes based upon the criteria described above. Six putative genes were shown to be missing an identifiable MHC class I C-terminal domain (Table 7).

## Discussion

The work described in this study clarifies the physical map of the sheep MHC class I region and provides an updated gene annotation. It is important for future studies that a reliable map and gene content of the MHC is available. This study details the gene content and arrangement within the class I region obtained by sub-cloning and sequencing of CHORI BACs that contain class I sequences. In addition, we used extensive manual, rather than automated, gene prediction analyses to determine the identity and location of MHC genes in the Class I region within Chinese Merino BACs previously published by Gao, et al. [30]. These analyses provide a more detailed description of gene content within the sheep MHC class I region and allows a comparison between the currently available genome sequence data in the NCBI database to be performed. This work also clarifies ambiguous information related to the MHC Class I region that is available to date for public access; this will be of use to other researchers with an interest in the sheep MHC class I region and is essential for future targeted next generational re-sequencing of the MHC and fine-mapping the causal mutations for disease susceptibility.

Re-assembly of the BAC sequences used in the previously published Chinese Merino map together with annotation of the genes within was a necessary step to enable comparison with the CHORI BAC based clones mapped in this study. Gao and colleagues reported the location of each gene within the Chinese Merino map relative to their reportedly contiguous map; however the complete sequence map was not published in a public database [30]. Instead, the individual BAC sequences used for construction of the Chinese Merino map were published without identifying the overlapping regions between BAC sequences [30]. Such overlaps would have enabled confirmation of the final contiguous architecture reported by this group [30].

Assembly of the Chinese Merino BAC sequences with Geneious resulted in five contigs rather than the single long contig reported by Gao et al. [30]. These multiple contigs indicate the probable presence of gaps in the map inferred by Gao and colleagues. Analysis of overlapping regions between the Chinese Merino BAC sequences using manual methods (combination of BLAST, dotplot, genomic sequence alignment and various gene prediction programs) suggested that there are actually six contigs in the sheep MHC map published by Gao et al. [30]. Three gaps are present in the class I region while the remaining two gaps were identified in the class IIa region. The comparison of alignments generated by Geneious and a manual method suggests that the latter produced a better contig tiling path. This may be explained by the low sensitivity of Geneious for a sequence containing a string of undefined nucleotides (N), possibly resulting in omission of the complete BAC sequence from the assembly. It seems that Geneious is not always capable of discriminating between real contigs and false positives. For instance, Geneious failed to include four BAC sequences [GenBank: FJ985852, GenBank: FJ985862, GenBank: FJ985865 and GenBank: FJ985867] in the contig tiling path and indicated that there is an overlap between GenBank: FJ985854 and GenBank: FJ985864. Closer manual examination of the overlapping region between GenBank: FJ985854 and GenBank: FJ985864 revealed that there is a potential gap in this region, which encompasses a region between *TRIM26* and *BASP1* (*NAP22)*. The initial 764 bp in the 5′ end of BAC sequence GenBank: FJ985864 does not align with the 3′ overlapping region of GenBank: FJ985854, but aligns from bp 765 onwards. BLAST analysis indicates that the 764 bp sequence at the 5′ end of BAC GenBank: FJ985864 does not show a contiguous alignment with the downstream BAC sequence when examining the matching alignments with two BAC sequences from cattle. Instead this region shows a match with a region elsewhere in the same cattle BAC sequence. Self dotplot and BLAST analysis of GenBank: FJ985864 indicates an alignment of bp 1–359 approximately 26,000 downstream and bp 403–764 approximately 35,500 bp downstream, with an intervening string of undefined nucleotides between bp 360–402. Dotplot alignment with the sheep reference chromosome 20 also indicates two alignments 5′ of the main diagonal separated by approximately 9000 bp. The 764 bp sequence in the 5′ end of BAC GenBank: FJ985864 could possibly be due to a mistake introduced in the initial re-assembly of the BAC sequence. Each of the 26 BAC sequences present in the Chinese Merino map was sequenced through a DNA shotgun sequencing method, which involves sub-cloning, sequencing and assembling randomised 0.5–2.0 kbp small fragments of DNA to form a full-length BAC sequence [30]. If the 764 bp ambiguity is not present on BAC GenBank: FJ985864, the rest of the BAC would align with no gap with BAC GenBank: FJ985854. Despite the differences in the result produced by Geneious and the manual method, both showed that the MHC map published by Gao et al. [30] is not contiguous and appears incomplete.

The BAC sequence GenBank: FJ985873 does not overlap with either GenBank: FJ985864 or GenBank: FJ985870 and the gap size in this region relative to the cattle reference sequence map is expected to be approximately 150 kbp, however dotplot alignment of the BACs with the sheep reference chromosome 20 indicates the gap is more likely to be approximately 50 kbp. It is likely that there are several genes missing in this region due to the gap. Within the sheep reference chromosome 20 there are two MHC Class I like genes and an envelope glycoprotein-like gene predicted in the region of the gap. Comparison of the class I region in other species suggests that the gap region may account for at least one peptide-presenting MHC class I gene [1, 23]. The gap between the *OVAR-MHCI* and *TRIM39* loci is notable. The other gap in the class I region is located between GenBank: FJ985875 and GenBank: FJ985859, which is between *EEF1A1* and an adjacent *OVAR-MHCI* locus. The size of this gap may be only a few thousand bp relative to the cattle map but the actual size is not known. A direct comparison to the sheep reference genome in this region is not possible because there are indels observed in the alignments between both GenBank: FJ985875 and GenBank: FJ985859 with the sheep reference sequence, indicating an inserted region of nucleotides in the sheep genome analysed by Gao et al. [30] or a deleted (or missing) region in the sheep reference genome. Misassembly in this region of one or both genomes is another possibility. The size of the gap between GenBank: FJ985867 and GenBank: FJ985862 and the genes at either end of the gap is not known because the class IIa region is yet to be annotated. The size of the gap in the class IIa region between GenBank: FJ985876 and GenBank: FJ985865 is also unknown.

Sequencing and re-assembly of CHORI BAC sub-clones provided a low resolution physical map of two separate areas spanning approximately 436 kbp within the class I region. Identification of ten new genes in this study adds significantly to the incomplete annotation in the original Chinese Merino map. These ten genes account for approximately 14 % of gene content within the class I region relative to the cattle reference map. The genes identified in CHORI BAC sequences are also present in the class I region of other mammals such as cattle, horse, human and pig [1, 23, 39–41]. The sheep MHC map derived from Chinese Merino published by Gao et al. [30], predicted 22 orthologous genes that have yet to be mapped to the cattle MHC.

In contrast, annotation of genes within the BAC sequences reported above for the class I region showed that there was a high level of sequence identity between genes within the Chinese Merino BAC sequences and known genes previously reported in sheep (*Ovis aries*) and cattle (*Bos taurus*). Amongst the 68 genes predicted in this study using the Chinese Merino BAC sequences, 38 (~56 %) were reported by Gao et al. [30]. Conversely, and not taking into consideration novel and predicted genes, of the 47 genes reported by Gao et al. [30] in the Class I region, 9 were not identified in this study. The ten genes identified in the CHORI BAC sequences were confirmed through re-analysis of the Chinese Merino BAC sequences, indicating that the gene prediction methods (BLAST and Ensembl pipeline) used by Gao et al. [30] were not entirely accurate.

Our revised map of the MHC Class I region annotates 65 genes, from *GABBR1* to *MCCD1* in a telomeric to centromeric direction, and represents a consensus map taking into consideration our re-annotation of the Chinese Merino BACs, the sheep reference chromosome 20 and the cattle reference chromosome 23. Fifty two of the annotated genes have been identified in both the Chinese Merino BACs and the sheep reference chromosome 20, nine genes have been identified in the Chinese Merino BACs, but not the sheep reference chromosome 20 and four genes are present in the sheep reference chromosome 20 but not identified in the Chinese Merino BACs. The four genes not identified in the Chinese Merino BACs are all located in indel regions identified by dot plot sequence alignment (see "Re-analysis of Chinese Merino MHC contig map" in Results section). A *ribosome production factor 2 homolog (RPF2)-like* gene (*LOC101111233*) is annotated between *TRIM26* and *LOC101110973* (*BASP1-like*) on the sheep reference chromosome 20. *LOC101111233* is annotated within an apparent insertion of 3934 bp in the reference chromosome 20 sequence that is not present in the corresponding Chinese Merino BAC sequence, as indicated in an alignment with BAC GenBank: FJ985864. The gap is located ~8000 bp from the 5′ end of the reverse complemented GenBank: FJ985864 sequence (see Additional file 2: Figure S3). RPF2 has not previously been identified in the MHC Class I region in other closely related species. The gene is annotated on chromosome 9, 10 and 1 in cattle, house mouse and pig, respectively. RPF2 has also been annotated within the sheep reference genome on chromosome 8. The structure of the two genes differs markedly; however, the translated protein sequences are identical. The mRNAs differ by only one bp in their corresponding sequences; however, the gene located on chromosome 8 has longer 5′ and 3′ untranslated regions annotated. The *RPF2* on chromosome 8 in sheep is annotated on the reverse complement strand and has ten exons; the exon size and distribution are the same as that annotated for the gene in the cattle, mouse, human and pig genomes. Genes 5′ on the same strand are *general transcription factor IIIC, polypeptide 6 (GTF3C6)* and *adenosylmethionine decarboxylase 1 (AMDI)*. Genes 3′ on the same strand are *solute carrier family 16, member 10 (aromatic amino acid transporter) (SLC16A10)* and *KIAA1919*. This is the same gene order seen in the cattle, mouse and human genomes. In the pig genome, *GTF3C6* and *AMDI* are 5′ to *RPF2*, but *SLC16A10* and *KIAA1919* have not been annotated 3′ to the gene. *LOC101111233* is annotated on the forward strand with two exons. Based on an alignment of mRNA and genomic sequence from the two genes, the first exon of *LOC101111233* appears to be a concatenation of the first nine exons of *RPF2*, whereas the intron and second exon correspond to the ninth intron and tenth exon of *RPF2* (data not shown). Due to this altered gene structure, we suggest that *LOC101111233* may be the result of a gene duplication event involving retrotransposition. This may represent a breed specific gene duplication, which will require further investigation to clarify. Three genes have been identified in the sheep reference chromosome 20 within the apparent gap region between Chinese Merino BACs GenBank: FJ985870 and GenBank: FJ985873. These include an *envelope glycoprotein-like* gene (*LOC101108432*) and two *MHC Class I-like* genes. One of the nine genes identified in the Chinese Merino BACs but not annotated on sheep reference chromosome 20 - C20H6orf12 - was found in a region of high sequence similarity between BAC GenBank: FJ985869 and the sheep reference sequence. Analysis of this region on sheep chromosome 20 using FGENESH+ with the predicted *C20H6orf12* as homolog indicates that the gene is present, and lies between genes *ZFP57* and *ZNRD1* on the forward strand (data not shown). The remaining eight genes in our map that are not annotated in the sheep reference sequence occur in sequence that is present in GenBank: FJ985875 but not in the sheep reference chromosome 20. Genes found in this region of GenBank: FJ985875 include one *DPCR1-like* gene, three *Mucin-like* genes, three *MHC Class I-like* genes and *eukaryotic translation elongation factor 1 alpha 1 (EEF1A1)*. Of these, only *EEF1A1* has not been annotated in the MHC Class I region in other closely related species. A search of the NCBI databases indicates that *EEF1A1* partial mRNAs have been isolated in sheep, but the gene has not been mapped to a chromosome to date. The gene is annotated on chromosome 9 in both cattle and house mouse, chromosome 8 in rats and chromosome 6 in humans but outside of the MHC region. BLAST analysis of the *EEF1A1* gene sequence from GenBank: FJ985875 against the sheep genome on the UCSC genome browser revealed near continous alignments with 99.9 % identity on chromosomes 4, 6 and 22. *EEF1A1* RefSeq mRNAs from a number of species map to the same regions on these chromosomes (data not shown). More broken alignments matching exon regions from *EEF1A1* annotation tracks from other species were observed on chromosomes 1, 2, 8, 10 and 11 (data not shown). It was noted that *EEF1A1* RefSeq mRNAs from cattle, mouse, rat and humans all mapped to the alignment on sheep chromosome 8; eight exons appear to be present in this region in these species. This would appear to be the most likely location for the functional gene in sheep. An alignment on chromosome 20 in a region matching annotation tracks from *EEF1A1* genes in other species was not observed. Sequencing of additional sheep genomes from various breeds is required to determine the accuracy of the current assemblies.

The revised sequence annotation shows that the general structure and gene content in the sheep MHC class I region is more similar to that of other mammals than previously suggested. There is however, a slight difference in the actual gene arrangement [1, 23, 39–42]. The class I genes involved in peptide presentation in some mammalian species such as chimpanzee [43], human [44], rhesus macaque [45] and horse [41], are often clustered within three distinct locations designated as the alpha (between *MOG* and *PPP1R11*), beta (between *POU5F1* and *BAT1*, which borders the class III region) and kappa (between *TRIM26* and *GNL1*) blocks. However, the clustering of peptide-presenting MHC class I genes in sheep does not fit entirely into the alpha, beta and kappa block framework. In a previous study of the sheep MHC, the presence of an additional novel block located between *GTF2H4* and *CDSN* was suggested [17]. Analysis of gene organisation within the class I region in this study confirms the presence of such a novel block. In this study, there are at least two definite MHC class I and two MHC class I-like genes between *GTF2H4* and *CDSN*. The exact number of peptide-presenting MHC class I genes is not known due to the presence of a gap in this block. In addition, this study reveals that there is no evidence for the presence of peptide-presenting MHC class I genes between *MOG* and *PPP1R1* (alpha block) as reported in other organisms. This finding is also in agreement with the previously reported sheep MHC study by Liu et al. [17]. The blocks of peptide-presenting genes are separated by numerous other class I genes with immune and non-immune related functions. The organisation of other sheep genes in the class I region is similar to the closely related cattle MHC.

The organisation of MHC class I peptide-presenting genes in distinct blocks, which are interspersed between other genes located within the class I region, is most likely due to segment or tandem block duplication [46–48]. The framework hypothesis suggests that the MHC class I region is a “conserved ordered segment” that represents a dense region of genes with essential functions, whose alterations are deleterious [42].

This study has assembled a single haplotype and shown that it is more similar to the reference cattle sequence. However, there is considerable diversity among MHC haplotypes in other species [49, 50]. Therefore additional haplotypes will need to be sequenced and assembled to provide a true picture of MHC diversity and structural evolution.

## Conclusion

The analysis performed in this study updates the existing sheep MHC map and enhances annotation of the genes present in the MHC class I region. This study also provides useful knowledge to complement the publicly available sequence information on NCBI regarding the Chinese Merino BACs, so that the information can be easily interpreted for future studies. In particular, the telomeric to centromeric orientation of BACs used by Gao and colleagues [30] has been resolved, overlapping sequence regions identified, gaps in the sheep MHC class I map mapped and the putative position of loci within each BAC encompassing MHC class I region detailed.

The updated sequence map provides a reference for future studies and will simplify the use of next generation sequences and SNP chips for multiple MHC studies including determination of the gene/genes responsible for resistance to infectious and parasitic diseases.

## Methods

### Sub-cloning of BAC DNA and sequencing

Three BAC clones (CHORI 243–269 M18, CHORI 243-390H16 and CHORI 243–454E19) derived from a ram of the Texel breed were used. These BAC clones had been previously shown to contain MHC class I sequence by J. Qin/D.Groth (unpublished 2006). DNA was extracted using the standard protocol of the QIAGEN® Large-Construct Kit. BAC DNA isolated from each of the clones was digested with *Pst I* restriction enzyme (Promega) and sub-cloned into the *Pst I* site of the pGEM® plasmid vector. BAC DNA was digested in a 10 μL reaction consisting of 8 μL of BAC DNA (500 ng – 1000 ng), 1 μL of restriction enzyme (10 units) and 1 μL of restriction enzyme buffer (10X). The pGEM® vector was digested in a 25 μL reaction as follows; 20 μL of vector (5 μg), 3 μL of restriction enzyme (30 units) and 2 μL of restriction enzyme buffer (10X). The mixture was mixed gently and incubated at 37 °C for 2–3 h, followed by heat inactivation of the restriction enzyme at 65 °C for 15 min. Restriction enzyme digested vector was treated with modifying enzyme Shrimp Alkaline Phosphatase (SAP) (Promega) to catalyse dephosphorylation of 5′ phosphates from the pGEM® vector. The reaction mix for SAP treatment contained vectors digested with restriction enzyme (5 μg), 10x SAP buffer and SAP enzyme (5 units). The mixture was incubated at 37 °C for 30 min and subsequently heat-denatured at 65 °C for 15 min. The ligation reaction of 10 μL volume was prepared as follows: 3 μL of restriction enzyme digested BAC DNA was mixed with 1 μL SAP treated pGEM® -3Z vector (50 ng), 4 μL sterile water, 1 μL 10x ligase buffer and 1 μL T4 DNA ligase (3 units). The reaction was incubated for 15 h at 14 °C to increase the number of transformants. Transformation of ligated recombinant vector into ELECTROMAX™ DH5α-ETM *E.coli* cells was performed by an electroporation method using a Biorad Gene Pulser II. Immediately after electroporation, the transformation mixture was incubated in 700 μL of SOC medium at 37 °C for 1 h with gentle shaking. The transformation mixture (100 μL) was plated onto LB agar plates supplemented with 0.1 mg/mL of ampicillin, 0.1 mM of IPTG and 40 μg/mL of X-GAL and incubated inverted overnight at 37 °C. Forty to fifty random recombinant clones from each BAC were then purified using the standard protocol of the AxyprepTM Plasmid Miniprep Kit (Axygen) and sequenced using the standard universal M13 forward and reverse primers, to ensure good quality double pass sequences were obtained. DNA sequencing was performed by Macrogen Inc. (Korea) on an ABI 3730XL sequencer. Internal primers were used when sequencing DNA fragments larger than 1000 bp to ensure contiguous and reliable sequence. The average size of these clones was 800–1000 bp, with an average size of the BACs of 200 kbp (390H16 and 454E19) and 150 kbp (269 M18). This represents an approximate 25–30 % coverage of each BAC. Concentration of the templates and primers submitted to Macrogen Inc was 10 ng/μL and 5 pmol/μL respectively.

### Analysis of CHORI BAC sub-clones

CHORI BAC sequences were screened and corrected for vector contamination using the Vecscreen program on the National Center for Biotechnology Information (NCBI) website. Vector NTI® software (default settings) was used to check sequence quality and assemble contigs. NCBI BLAST was used to determine the location of CHORI BAC contig sequences relative to the cattle reference genome sequence [GenBank: AC_000180]. The cattle genome was used as a reference for mapping the sequences because the cattle map is the most thoroughly curated ruminant map available in the NCBI database. NCBI BLAST was also used to determine the location of CHORI BAC contig sequences relative to the recently available sheep reference genome sequence [GenBank: NC_019477].

### Re-analysis of Chinese Merino sheep MHC map

In order to compare the MHC class I physical map constructed from CHORI BAC in this study with the Chinese Merino MHC map [30], the BAC sequences representing the Chinese Merino map were re-assembled as the contiguous sequence of the map has not been uploaded into a public database. Twenty BAC sequences representing the class I, IIa and III regions in the sheep MHC map proposed by Gao et al. (2010) were downloaded from NCBI and re-assembled using Geneious Pro 5.5 (Drummond et al. (2011) - unpublished) in an attempt to form a contiguous assembly. The BAC sequences used for this analysis were: FJ985852.1, FJ985853.1, FJ985854.1, FJ985856.1, FJ985857.1, FJ985859.1, FJ985861.1, FJ985862.1, FJ985864.1, FJ985865.1, FJ985866.1, FJ985867.1, FJ985868.1, FJ985869.1, FJ985870.1, FJ985872.1, FJ985873.1, FJ985874.1, FJ985875.1 and FJ985876.1. The assembly was compared to the published Chinese Merino sheep MHC map [30]. Further analyses were performed to identify discrepancies between the published map and the result we obtained from assembly of BAC sequences using the following strategy: Potential overlaps between BAC sequences were determined using the NCBI BLAST option to align two sequences (http://blast.ncbi.nlm.nih.gov/Blast.cgi). Sequences that overlapped at the 5′ or 3′ ends were subsequently aligned with the CHAOS/DIALIGN software [51] provided at http://dialign.gobics.de/chaos-dialign-submission. Alignments were examined and edited, where required, using Seaview 4.2.12 [52] to provide an optimal alignment and determine overlap boundaries. Thirteen of the BAC sequences examined were reverse complemented before pairwise sequence alignment in order to provide a contiguous assembly in a telomeric to centromeric direction [30].

Ten of the Chinese Merino BAC sequences [30] proposed to cover the MHC class I region were further analysed for gene content, these were: FJ985869, FJ985854, FJ985864, FJ985870, FJ985873, FJ985868, FJ985852, FJ985875, FJ985859 and FJ985874. Of the ten sequences, seven required reverse complementation before analysis in order to facilitate mapping in a contiguous 5′ to 3′ direction. Gene content analysis was performed as follows: BAC sequences were masked for repeats with Repeatmasker, open version 3.2.9, then analysed with GENSCAN (http://genes.mit.edu/GENSCAN.html) and Softberry FGENESH (http://linux1.softberry.com/all.htm). Predicted transcripts were submitted to the NCBI BLAST server to identity putative gene transcripts by homology to known genes previously reported in mammalian species, in particular *Ovis aries* or *Bos taurus*. To refine predictions for putative genes, BAC sequences were subsequently analysed with FGENESH+ using one or more of the best matching proteins as a homologue. *Bos taurus* was chosen as the model organism for both FGENESH and FGENESH+ and up to five variant transcripts were considered. In the case where there appeared to be multiple copies of the same or a similar gene in a single BAC, FGENESH+ gene prediction was localised to each particular region of interest. The most suitable transcript for each gene was selected based on alignment with known genes from *Ovis aries* (when available), *Bos taurus*, *Sus scrofa* and *Homo sapiens*.

### Sequence comparison of Chinese Merino BACs with MHC Class I region of reference sheep chromosome 20

Sequence covering the MHC Class I region in the sheep reference genome was downloaded from the NCBI Genbank database [GenBank: NC_019477; region: 26884456….28150000) and aligned with Chinese Merino BACs FJ985869.1, FJ985854.1, FJ985864.1, FJ985870.1, FJ985873.1, FJ985868.1, FJ985852.1, FJ985875.1, FJ985859.1 and FJ985874.1 [30] using the dot plot analysis program GEnome PAir - Rapid Dotter (Gepard) version 1.3 [53]. Word length was set to 30. All other parameter settings were left at default values. Chinese Merino BAC sequences were aligned in a telomeric to centromeric direction in order to preserve a consistent orientation in the dot plots. Large indels and other mis-alignments discovered in the dot plots were further investigated using NCBI BLAST with parameter settings for MEGABLAST (data not shown).

### Comparative analysis of MHC Class I maps

The NCBI BLAST program was used with default settings for MEGABLAST to align the CHORI BAC end sequences to the sheep and cattle reference sequences [GenBank: AC_000180 and GenBank:NC_019477] and Chinese Merino BAC sequences in order to determine the boundaries of each CHORI BAC within the MHC Class I region of all maps. NCBI BLAST (MEGABLAST) was also used to align both the CHORI BAC sequences and Chinese Merino BAC sequences to the sheep reference genome chromosome 20 [GenBank: NC_019477] in order to verify the location and identity of MHC Class I genes within each BAC contig map.

### Identification of functional domains in MHC class I histocompatibility antigen-like proteins

Predicted MHC class I histocompatibility antigen proteins were checked for the presence of a signal peptide (leader sequence) using the SignalP 4 server [54] at URL http://www.cbs.dtu.dk/services/SignalP/. Predicted proteins were screened for the presence of MHC class I domains using the NCBI Conserved Domain Database [55] at URL http://www.ncbi.nlm.nih.gov/Structure/bwrpsb/bwrpsb.cgi and Pfam [56] at URL http://pfam.sanger.ac.uk/. The Pfam database proved more sensitive for detection of the cytoplasmic (CP) domain. Transmembrane (TM) domain was predicted using the TmPred program [57] provided by EMBnet (http://www.ch.embnet.org/software/TMPRED_form.html).

## Additional files

Additional file 1:
**Pairwise alignment between BAC sequences representing the Chinese Merino map.** Description of data: Additional file 1 is a document listing a series of dotplot analyses of the Chinese Merino BAC sequences published in the NCBI database by Gao et al. (2010), along with the interpretation of the analyses. (DOCX 1249 kb) Additional file 2:
**Detailed Gepard analysis and comparison between Chinese Merino BACs and sheep reference chromosome 20.** Description of data: Additional file 2 is a figure illustrating the dotplot analysis between Chinese Merino BAC sequences published by Gao et. al. (2010) and sheep reference chromosome 20 [GenBank: NC_190477 Region: 26884456–28150000]. (DOCX 654 kb) Additional file 3:
**List of genes in the MHC Class I region of sheep and cattle reference genome.** Description of data: Additional file 3 is a document listing the gene name, symbol and description of genes located in the MHC Class I region in sheep [GenBank: NC_019477.1] and cattle [GenBank: AC_000180.1]. (DOCX 43 kb) Additional file 4:
**Amino acid sequence alignments of MHC class I genes.** Description of data: Additional file 4 shows amino acid sequence alignments of MHC class I genes from selected BAC sequences published by Gao et al., 2010 and the primary sheep reference assembly for chromosome 20 (NC_019477.1) in comparison with six class I reference sequences from the IPD database. (DOCX 491 kb)
