# Supplementary material for: A comprehensive mapping of the structure and gene organisation in the sheep MHC class I region
Source: BMC Genomics. 2015 Oct 19;16:810. doi: 10.1186/s12864-015-1992-4 (PMC4613773; doi:10.1186/s12864-015-1992-4)

**Additional data file 1**

A series of dotplots generated using the NCBI BLAST program illustrating the results of pairwise alignment between BAC sequences representing the Chinese Merino map is shown in Supplementary Figure 1. The 5’ end of GenBank: FJ985869 has a significant overlap with the 3’ end of GenBank: FJ985854 in a plus/plus orientation. The 5’ end of GenBank: FJ985854 has a short but significant overlap near the 3’ end of GenBank: FJ985864 in a plus/plus orientation; however, the alignment ends at bp 141596 instead of at the 3’ end of the sequence (142360) as would be expected in a contiguous assembly as indicated in the Chinese Merino map. There is no significant overlap between GenBank: FJ985873 and either GenBank: FJ985864 or GenBank: FJ985870, contrary to what is shown in the Chinese Merino map. The 5’ end of GenBank: FJ985864 (1- ~116,000) overlaps with the 5’ end (1-~116,000) of GenBank: FJ985870 in a plus/minus orientation. The 3’ end of GenBank: FJ985873 overlaps with the 3’ end of GenBank: FJ985868 in a plus/minus orientation. The 5’ end of GenBank: FJ985868 overlaps with the 3’ end of GenBank: FJ985852 in a plus/plus orientation. The 5’ end of GenBank: FJ985868 overlaps with the 3’ end of GenBank: FJ9858675 in a plus/plus orientation. The 5’ end of GenBank: FJ985852 overlaps with the 3’ end of GenBank: FJ9858675 in a plus/plus orientation. There is no significant overlap between GenBank: FJ985875 and GenBank: FJ985859, contrary to what is shown in the Chinese Merino map. The 5’ end of GenBank: FJ985859 overlaps with the 5’ end of GenBank: FJ985874 in a plus/minus orientation. The 3’ end of GenBank: FJ985874 overlaps with the 5’ end of GenBank: FJ985856 in a plus/plus orientation. The 3’ end of GenBank: FJ985856 overlaps with the 5’ end of GenBank: FJ985861 in a plus/plus orientation. The 3’ end of GenBank: FJ985861 overlaps with the 3’ end of GenBank: FJ985872. The orientation is plus/minus. The 5’ end of GenBank: FJ985872 overlaps with the 3’ end of GenBank: FJ985857 in a plus/plus orientation. The 5’ end of GenBank: FJ985857 overlaps with the 5’ end of GenBank: FJ985853 in a plus/minus orientation. The 3’ end of GenBank: FJ985853 overlaps with the 3’ end of GenBank: FJ985867 in a plus/minus orientation. There is no overlap between GenBank: FJ985867 and GenBank: FJ985862 contrary to what is shown in the Chinese Merino map. The 5’ end of GenBank: FJ985862 overlaps with the 3’ end of GenBank: FJ985866 in a plus/plus orientation. The 5’ end of GenBank: FJ985866 overlaps with the 3’ end of GenBank: FJ985876 in a plus/plus orientation. The 5’ end of GenBank: FJ985876 overlaps with the 3’ end of GenBank: FJ985865 in a plus/plus orientation. The overlap is dubious since there are several breaks in it. All alignments in the overlap region are on the order of 94% identity with numerous small indels and base substitutions. This is not likely to represent a true overlap of contigs, contrary to what is shown in the Chinese Merino map.

**Supplementary Figure 1:** Dot plots of BAC sequence contigs published in the NCBI database by Gao *et al*. (2010). X axis represents the first sequence named in the 5’- >3’ direction left to right. The y axis represents the second sequence in the 5’- >3’ direction from bottom to top. Unbroken diagonal lines in the corners of the axes are indicative of overlap regions between BAC sequences. Diagonals drawn up and to the right indicate alignments in the plus/plus orientation. Diagonals drawn down and to the right indicate alignments in the plus/minus orientation.


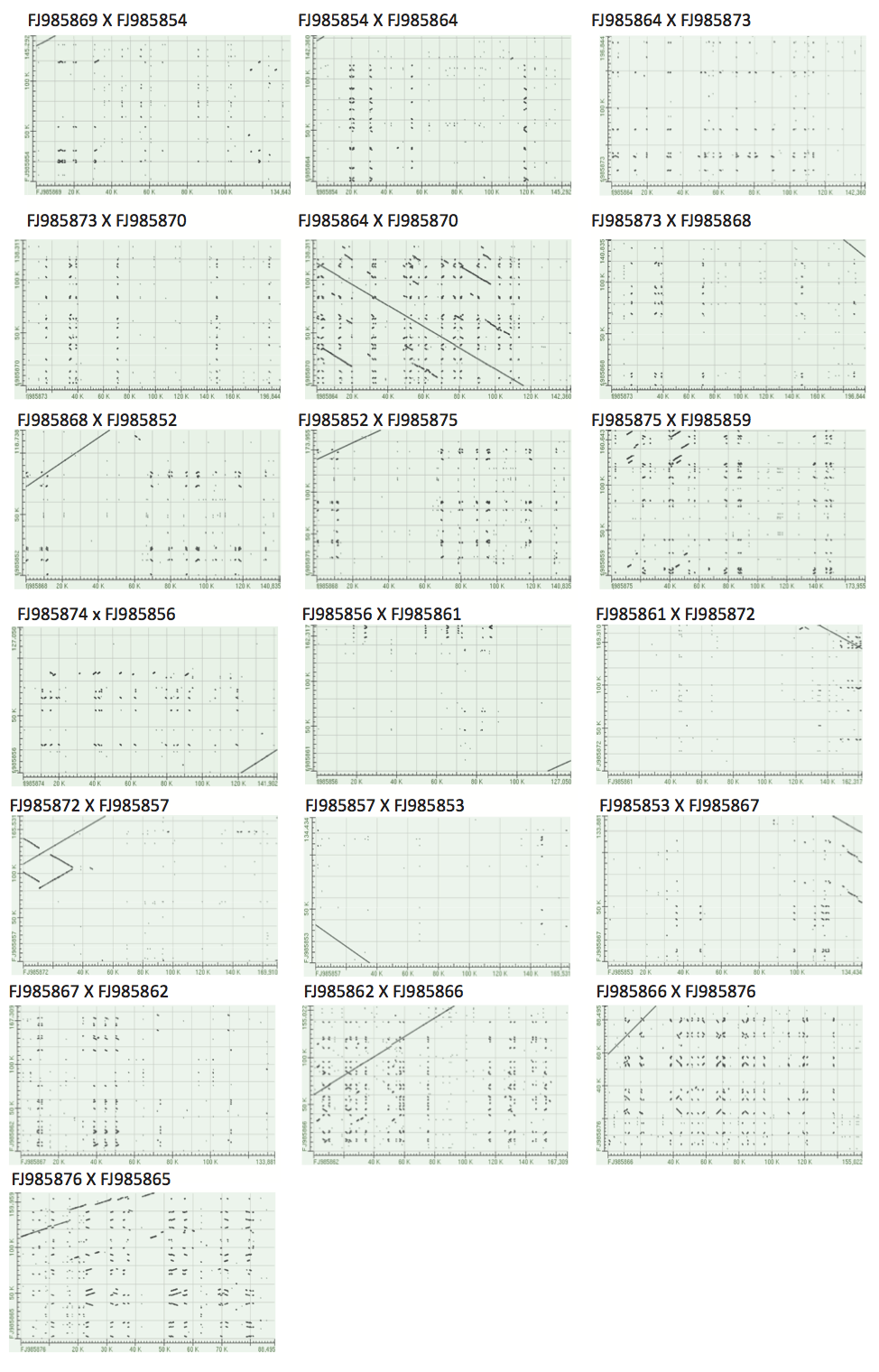


BAC sequences that were in 3’- 5’ orientation in the initial dotplots (Supplementary Figure 1) were reverse complemented and the dotplot analysis repeated for all of the BAC sequences published by *Gao et al*. (2010). Supplementary Figure 2 shows this series of dotplot analyses of the Chinese Merino BAC sequences in a 5’-3’ orientation, telomere to centromere. The 3’ end of GenBank: FJ985869_rc aligns with the 5’ end of GenBank: FJ985854_rc in a plus/plus orientation with an overlap of 10399 bp. The 3’ end of the reverse complement of GenBank: FJ985854 has a significant pairwise alignment near the 5’ end of the reverse complement of GenBank: FJ985864 in a plus/plus orientation, but the alignment does not begin until bp 765 in GenBank: FJ985864. This may represent a misassembly of the first 765 bp of GenBank: FJ985864. The 3’ end of the reverse complement of GenBank: FJ985864 aligns with the 5’ end of GenBank: FJ985870 in a plus/plus orientation with an overlap of 116126 bp. The 3’ end of GenBank: FJ985873 aligns with the 5’ end of the reverse complement of GenBank: FJ985868 in a plus/plus orientation with an overlap of 16758 bp. The 3’ end of the reverse complement of GenBank: FJ985868 aligns with the 5’ end of the reverse complement of GenBank: FJ985852 in a plus/plus orientation with an overlap of 46,198 bp. The 3’ end of the reverse complement of GenBank: FJ985868 aligns with the 5’ end of the reverse complement of GenBank: FJ9858675 in a plus/plus orientation with an overlap of 35324 bp. The 3’ end of the reverse complement of GenBank: FJ985852 aligns with the 5’ end of the reverse complement of GenBank: FJ9858675 in a plus/plus orientation with an overlap of 108,231 bp. The 3’ end of the reverse complement of GenBank: FJ985859 aligns with the 5’end of GenBank: FJ985874 in a plus/plus orientation with an overlap of 18,619 bp. The 3’ end of GenBank: FJ985874 aligns with the 5’ end of GenBank: FJ985856 in a plus/plus orientation with an overlap of 20,418 bp. The 3’ end of GenBank: FJ985856 aligns with the 5’ end of GenBank: FJ985861 in a plus/plus orientation with an overlap of 11759 bp. The 3’ end of GenBank: FJ985861 aligns with the 5’ end of the reverse complement of GenBank: FJ985872 in a plus/plus orientation with an overlap of 27705 bp. The 3’ end of the reverse complement of GenBank: FJ985872 aligns with the 5’ end of the reverse complement of GenBank: FJ985857 in a plus/plus orientation with an overlap of 55107 bp. The 3’ end of the reverse complement of GenBank: FJ985857 aligns with the 5’ end of GenBank: FJ985853 in a plus/plus orientation with an overlap of 35410 bp. The 3’ end of GenBank: FJ985853 aligns with the 5’ end of the reverse complement of GenBank: FJ985867 in a plus/plus orientation with an overlap of 15709 bp. The 3’ end of the reverse complement of GenBank: FJ985862 aligns with the 5’ end of the reverse complement of GenBank: FJ985866 in a plus/plus orientation with an overlap of 94777 bp. The 3’ end of the reverse complement of GenBank: FJ985866 aligns with the 5’ end of the reverse complement of GenBank: FJ985876 in a plus/plus orientation with an overlap of 29542 bp. The 3’ end of the reverse complement of GenBank: FJ985876 aligns with the 5’ end of the reverse complement of GenBank: FJ985865 in a plus/plus orientation. However, there are several large indels in the diagonal region of overlap. All pairwise alignments in the overlap diagonal share only approximately 94% identity and show a number of small indels and substitutions.

**Supplementary Figure 2:** Dot plots of the Chinese Merino BAC sequence contigs in a telomeric to centromeric (5’ to 3’) orientation. Dotplots with no potential overlap are not shown in this figure. The text ‘rc’ appended to the accession number indicates the sequence was reverse complemented before alignment.


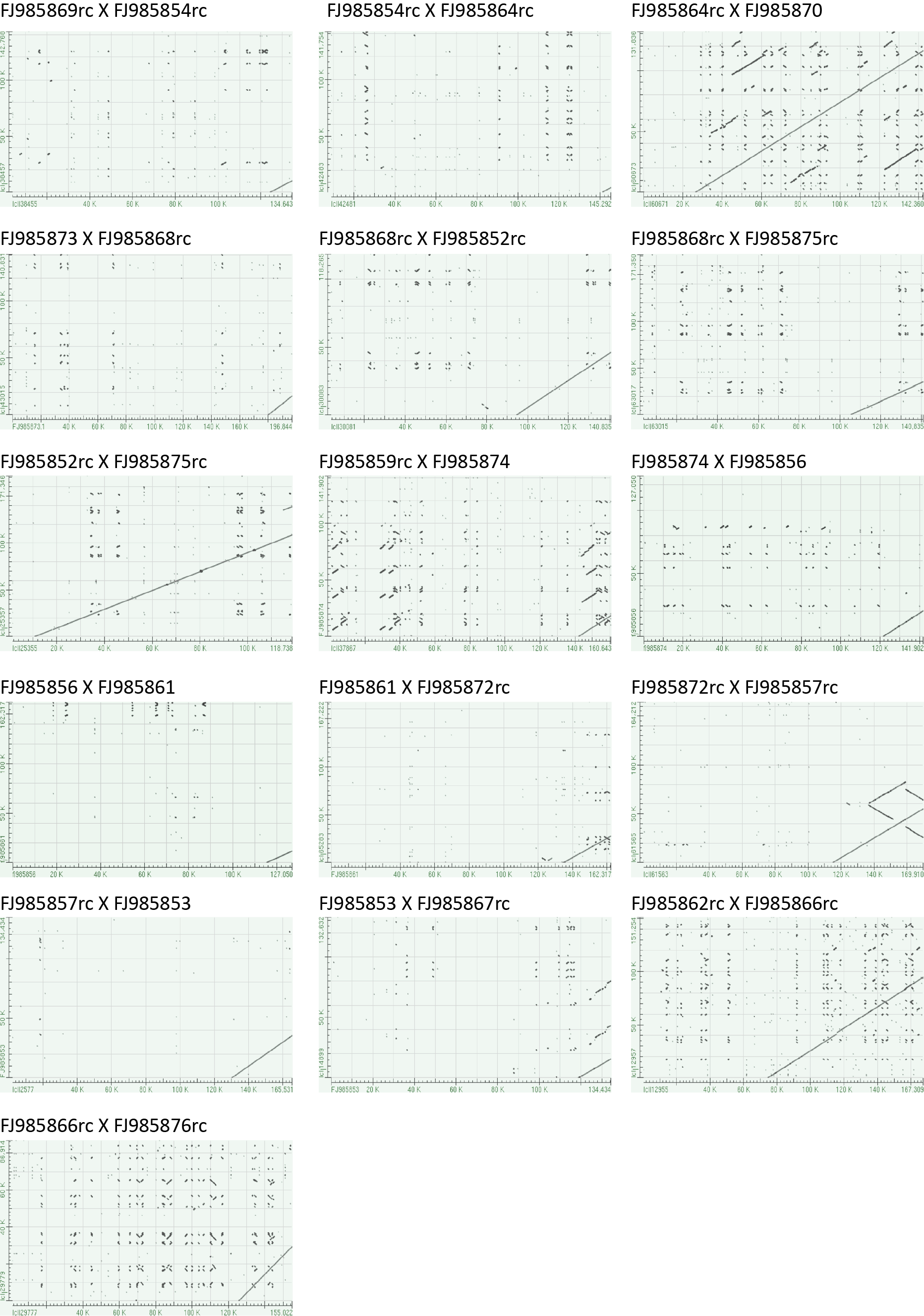

Supplement: Additional file 1: — Pairwise alignment between BAC sequences representing the Chinese Merino map. Description of data: Additional file 1 is a document listing a series of dotplot analyses of the Chinese Merino BAC sequences published in the NCBI database by Gao et al. (2010), along with the interpretation of the analyses. (DOCX 1249 kb) [file 12864_2015_1992_MOESM1_ESM.docx]
