# Supplementary material for: A comprehensive mapping of the structure and gene organisation in the sheep MHC class I region
Source: BMC Genomics. 2015 Oct 19;16:810. doi: 10.1186/s12864-015-1992-4 (PMC4613773; doi:10.1186/s12864-015-1992-4)

**Additional data file 2**

**Supplementary Figure 3:** Series of dotplot alignments constructed with Gepard v1.3 between Chinese Merino BACs published by Gao, *et. al*. (2010) and sheep reference chromosome 20 [GenBank: NC_190477; region: 26884456-28150000]. Alignments were drawn to the same scale to allow for direct comparison. Alignments were constructed in a telomeric to centromeric direction. Seven of the BAC sequences were reverse complemented prior to analysis. The orientation of the BACs was from telomere to centromere. Chromosome 20 from the sheep reference genome is in the opposite orientation, hence the alignment diagonals run from bottom left to top right.


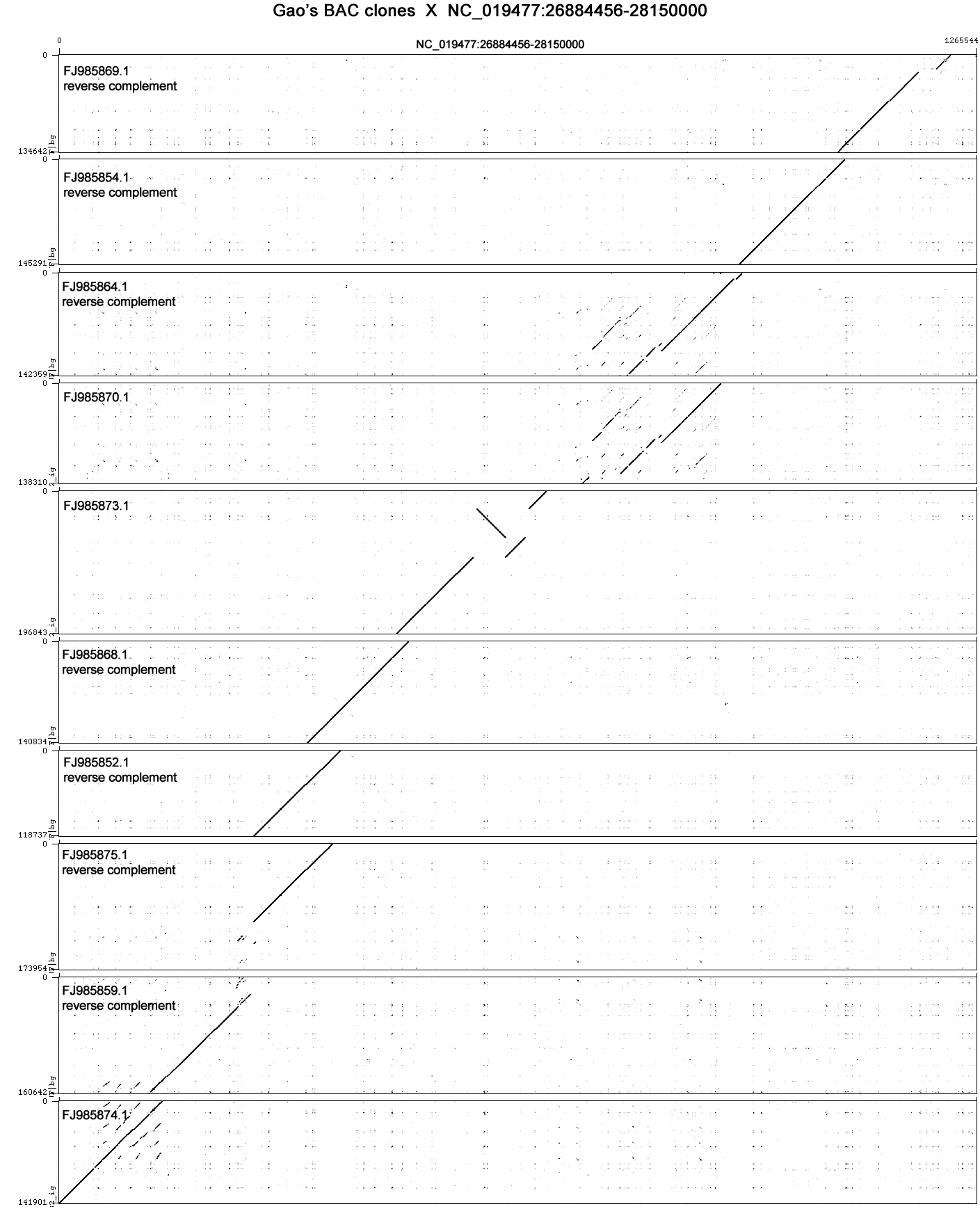

Supplement: Additional file 2: — Detailed Gepard analysis and comparison between Chinese Merino BACs and sheep reference chromosome 20. Description of data: Additional file 2 is a figure illustrating the dotplot analysis between Chinese Merino BAC sequences published by Gao et. al. (2010) and sheep reference chromosome 20 [GenBank: NC_190477 Region: 26884456–28150000]. (DOCX 654 kb) [file 12864_2015_1992_MOESM2_ESM.docx]
