# Supplementary material for: A comprehensive mapping of the structure and gene organisation in the sheep MHC class I region
Source: BMC Genomics. 2015 Oct 19;16:810. doi: 10.1186/s12864-015-1992-4 (PMC4613773; doi:10.1186/s12864-015-1992-4)
Supplement: Additional file 3: — List of genes in the MHC Class I region of sheep and cattle reference genome. Description of data: Additional file 3 is a document listing the gene name, symbol and description of genes located in the MHC Class I region in sheep [GenBank: NC_019477.1] and cattle [GenBank: AC_000180.1]. (DOCX 43 kb) [file 12864_2015_1992_MOESM3_ESM.docx]

**Additional data 3**

**Supplementary Table 1**: Sheep Chromosome 20 [GenBank: NC_019477.1] Annotated MHC Class 1 region from the NCBI Genome database (November 2014).

| **Gene Symbol** | **Gene Name*** | **Strand** | **Gene Description** | **Gene Type** | **Location** |
| --- | --- | --- | --- | --- | --- |
| *UBD-like* | LOC101113965 | + | *ubiquitin D-like* | protein coding | 28146612..28148999 |
| *OR2H1-like* | LOC101111325 | - | *olfactory receptor 2H1-like* | pseudo | c:28130885..28132050 |
| *UBD-like* | LOC101113705 | - | *ubiquitin D-like* | protein coding | c: 28111204..28113466 |
| *OR2I1-like* | LOC101111059 | + | *putative olfactory receptor 2I1-like* | pseudo | 28106832..28110259 |
| *OR2I1-like* | LOC101110800 | + | *putative olfactory receptor 2I1-like* | pseudo | 28097084..28097873 |
| *UBD-like* | LOC101110547 | - | *ubiquitin D-like* | protein coding | c: 28090849..28093435 |
| *C19orf12-like* | LOC101113442 | - | *protein C19orf12 homolog* | protein coding | c: 28068422..28079358 |
| *OR2I1-like* | LOC101110278 | + | *putative olfactory receptor 2I1-like* | pseudo | 28063720..28066582 |
| *GABBR1* |  | + | *gamma-aminobutyric acid (GABA) B receptor, 1* | protein coding | 28012311..28056210 |
| *MOG* |  | - | *myelin oligodendrocyte glycoprotein* | protein coding | c: 27997007..28008421 |
| *ZFP57* |  | + | *zinc finger protein 57 homolog (mouse)* | protein coding | 27992745..27997361 |
| *ZNRD1* |  | - | *zinc ribbon domain containing 1* | protein coding | c: 27972373..27975757 |
| *PPP1R11* |  | - | *protein phosphatase 1, regulatory (inhibitor) subunit 11* | protein coding | c:27964184..27967320 |
| *RNF39* |  | + | *ring finger protein 39* | protein coding | 27934906..27963376 |
| *TRNAL-CAA* |  | + | *transfer RNA leucine (anticodon CAA)* | tRNA | 27931958..27932039 |
| *TRIM31* |  | + | *tripartite motif containing 31* | protein coding | 27919516..27930157 |
| *TRIM40* |  | - | *tripartite motif containing 40* | protein coding | c:27880429..27891785 |
| *TRIM10* |  | + | *tripartite motif containing 10* | protein coding | 27868279..27876934 |
| *TRIM15* |  | - | *tripartite motif containing 15* | protein coding | c:27855293..27865217 |
| *TRIM26* |  | + | *tripartite motif containing 26* | protein coding | 27829509..27839079 |
| *RPF2-like* | LOC101111233 | + | *ribosome production factor 2 homolog* | protein coding | 27814318..27815849 |
| *BASP1-like* | LOC101110973 | + | *brain acid soluble protein 1-like* | pseudo | 27803500..27804406 |
| *OVAR-MHCI-like* | LOC101108963 | - | *BOLA class I histocompatibility antigen, alpha chain BL3-6-like* | pseudo | c:27766021..27796420 |
| *OVAR-MHCI-like* | LOC101110710 | - | *BOLA class I histocompatibility antigen, alpha chain BL3-7-like* | pseudo | c:27673673..27678484 |
| *TRIM26-like* | LOC101108697 | - | *tripartite motif-containing protein 26-like* | pseudo | c 27645827..27650044 |
| *LOC101108432* | LOC101108432 | + | *envelope glycoprotein-like* | protein coding | 27604155..27626210 |
| *OVAR-MHCI-like* | LOC101108171 | - | *BOLA class I histocompatibility antigen, alpha chain BL3-7-like* | pseudo | c:27596720..27602996 |
| *OVAR-MHCI* | LOC101107908 | + | *BOLA class I histocompatibility antigen, alpha chain BL3-7-like* | protein coding | 27566850..27573838 |
| *TRIM39-like* | LOC101106373 | - | *E3 ubiquitin-protein ligase TRIM39-like* | protein coding | c:27533628..27542131 |
| *ABCF1* |  | - | *ATP-binding cassette, sub-family F (GCN20), member 1* | protein coding | c:27505531..27518579 |
| *PRR3* |  | + | *proline rich 3* | protein coding | 27495427..27499481 |
| *GNL1* |  | - | *guanine nucleotide binding protein-like 1* | protein coding | c:27486991..27494543 |
| *RPP21* |  | + | *ribonuclease P/MRP 21kDa subunit* | protein coding | 27460755..27462368 |
| *PPP1R10* |  | + | *protein phosphatase 1, regulatory subunit 10* | protein coding | 27446468..27454847 |
| *MRPS18B* |  | - | *mitochondrial ribosomal protein S18B* | protein coding | c:27432758..27439029 |
| *ATAT1* |  | - | *alpha tubulin acetyltransferase 1* | protein coding | c:27419172..27431931 |
| *C20H6orf136* |  | - | *chromosome 20 open reading frame, human C6orf136* | protein coding | c:27415031..27418766 |
| *DHX16* |  | + | *DEAH (Asp-Glu-Ala-His) box polypeptide 16* | protein coding | 27402730..27414855 |
| *PPP1R18* |  | + | *protein phosphatase 1, regulatory subunit 18* | protein coding | 27391396..27399789 |
| *NRM* |  | + | *nurim (nuclear envelope membrane protein)* | protein coding | 27386533..27389389 |
| *MDC1* |  | + | *mediator of DNA-damage checkpoint 1* | protein coding | 27372265..27383751 |
| *TUBB2A* |  | - | *tubulin, beta 2A class IIa* | protein coding | c:27363398..27367892 |
| *FLOT1* |  | + | *flotillin 1* | protein coding | 27352471..27363092 |
| *IER3* |  | + | *immediate early response 3* | protein coding | 27350668..27351932 |
| *DDR1* |  | - | *discoidin domain receptor tyrosine kinase 1* | protein coding | c: 27239217..27250506 |
| *GTF2H4* |  | - | *general transcription factor IIH, polypeptide 4* | protein coding | c: 27223376..27231107 |
| *VARS2* |  | - | *valyl-tRNA synthetase 2, mitochondrial* | protein coding | 27210577..27223035 |
| *SFTA2* |  | + | *surfactant associated 2* | protein coding | 27206374..27207339 |
| *DPCR1-like* | LOC101106634 | - | *uncharacterized LOC101106634 (DPCR1-like)* | protein coding | c: 27187822..27204351 |
| *SAP30L-like* | LOC101109651 | - | *histone deacetylase complex subunit SAP30L-like* | pseudo | 27158375..27158905 |
| *OVAR-MHCI-like* | LOC101106374 | + | *BOLA class I histocompatibility antigen, alpha chain BL3-7-like (pseudogene – no transcript)* | pseudo | 27133720..27141901 |
| *C20H6orf15* |  | + | *chromosome 20 open reading frame, human C6orf15* | protein coding | 27108833..27109953 |
| *CDSN* |  | - | *corneodesmosin* | protein coding | 27100977..27105707 |
| *PSORS1C2* |  | + | *psoriasis susceptibility 1 candidate 2* | protein coding | 27083459..27084909 |
| *CCHCR1* |  | + | *coiled-coil alpha-helical rod protein 1* | protein coding | 27069757..27081419 |
| *TCF19* |  | - | *transcription factor 19* | protein coding | c:27065597..27069326 |
| *POU5F1* |  | + | *POU class 5 homeobox 1* | protein coding | 27059785..27064568 |
| *MIC-A* | LOC101105860 | - | *MHC class I polypeptide-related sequence A-like* | protein coding | c:27025994..27037088 |
| *OVAR-MHCI-like* | LOC101107641 | + | *BOLA class I histocompatibility antigen, alpha chain BL3-6-like* | pseudo | 27016488..27019600 |
| *OVAR-MHCI* | LOC101105609 | + | *BOLA class I histocompatibility antigen, alpha chain BL3-6-like* | protein coding | 26967605..26991453 |
| *OVAR-MHCI-like* | LOC101105367 | + | *BOLA class I histocompatibility antigen, alpha chain BL3-7-like* | pseudo | 26942864..26967556 |
| *IFITM3-like* | LOC101110456 | + | *interferon-induced transmembrane protein 3-like* | pseudo | 26941928..26942348 |
| *TSPAN3-like* | LOC101105113 | - | *tetraspanin-3-like* | pseudo | c:26930808..26931445 |
| *OVAR-MHCI†* | LOC101104866 | + | *HLA class I histocompatibility antigen, A-24 alpha chain-like* | protein coding | 26924140..26927680 |
| *MCCD1* |  | - | *mitochondrial coiled-coil domain 1* | protein coding | c:26897437..26898532 |
| *DDX39B* |  | + | *spliceosome RNA helicase DDX39B*  *(BAT1)* | protein coding | 26886115..26897170 |

*Gene Names only provided if different from Gene Symbol. † high similarity to bovine uncharacterised protein MGC126945

**Supplementary Table 2**: Bovine Chromosome 23 [GenBank: AC_000180.1] Annotated MHC Class 1 region from the NCBI Genome database.

| **Gene Symbol** | **Gene Name** | **Str** | **Gene Description** | **Gene Type** | **Location** |
| --- | --- | --- | --- | --- | --- |
| *OR2I1-like* | LOC101903658 | - | *putative olfactory receptor 2I1-like* | protein coding | c:28921606..28933993 |
| *UBD* |  | + | *ubiquitin D* |  | 28918428..28920748 |
| *OR2H1* |  | - | *olfactory receptor, family 2, subfamily H, member 1* |  | c:28902250..28918417 |
| *OR2H1-like* | LOC506486 | - | *olfactory receptor, family 2, subfamily H, member 1-like* |  | c:28888958..28892510 |
| *UBD-like* | LOC504548 | - | *ubiquitin D-like* |  | c:28873237..28875584 |
| *OR2G3-like* | LOC101903577 | + | *olfactory receptor 2G3-like* | pseudo | 28863423..28871851 |
| *C19orf12-like* | LOC786987 | - | *protein C19orf12 homolog-like* |  | c:28813918..28870287 |
| *OR2I1-like* | LOC100848480 | + | *putative olfactory receptor 2I1-like* | pseudo | 28809623..28812480 |
| *GABBR1* |  |  | *gamma-aminobutyric acid (GABA) B receptor, 1* |  | 28775569..28803896 |
| *LOC101906691* |  |  | *uncharacterized LOC101906691* | ncRNA | 28754175..28759750 |
| *MOG* |  | - | *myelin oligodendrocyte glycoprotein* |  | c:28738200..28752577 |
| *ZFP57* |  | + | *ZFP57 zinc finger protein* |  | 28718554..28739775 |
| *ZNRD1* |  | - | *zinc ribbon domain containing 1* |  | 28714104..28718047 |
| *PPP1R11* |  | - | *protein phosphatase 1, regulatory (inhibitor) subunit 11* |  | c:28707258..28710411 |
| *RNF39* |  | + | *ring finger protein 39* |  | 28701821..28707321 |
| *TRNAS-CGA* |  |  | *transfer RNA serine (anticodon CGA)* |  | 28678316..28678397 |
| *TRIM31* |  |  | *tripartite motif containing 31* |  | 28651502..28677457 |
| *TRIM40* |  |  | *tripartite motif containing 40* |  | 28625428..28638869, complement |
| *TRIM10* |  |  | *tripartite motif containing 10* |  | 28613833..28622421 |
| *TRIM15* |  |  | *tripartite motif containing 15* |  | 28600324..28611179, complement |
| *TRIM26* |  |  | *tripartite motif containing 26* |  | 28562997..28580855 |
| *TRIM26-like* | LOC526288 |  | *tripartite motif-containing 26 pseudogene* | pseudo | 28530732..28543769, complemen |
| *BOLA* |  |  | *MHC class I heavy chain* |  | 28502521..28506312, com |
| *RPL35AP* | LOC100295296 |  | *60S ribosomal protein L35a pseudogene* | pseudo | 28494840..28495272, com |
| *BOLA-JSP.1* |  |  | *MHC Class I JSP.1* |  | 28469733..28473374, com |
| *LOC100298822* |  |  | *uncharacterized LOC100298822* |  | 28425332..28450252, com |
| *BOLA-like* | LOC614091 |  | *BOLA class I histocompatibility antigen, alpha chain BL3-7-like* | pseudo | 28376891..28431461, com |
| *BOLA-NC1* |  |  | *non-classical MHC class I antigen* |  | 28351923..28358368, com |
| *BOLA-like* | LOC101905956 |  | *BOLA class I histocompatibility antigen, alpha chain BL3-7-like* | pseudo | 28330386..28334108, com |
| *BOLA* | LOC512672 |  | *major histocompatibility complex, class I* |  | 28299248..28311312 |
| *TOM20-like* | LOC101906024 |  | *mitochondrial import receptor subunit TOM20 homolog* | pseudo | 28297025..28298783, complement |
| *TRIM39* |  | - | *tripartite motif containing 39 (RPP21)* |  | 28257733..28273626, com |
| *GNL1* |  | + | *guanine nucleotide binding protein-like 1* |  | 28224593..28231252 |
| *PRR3* |  | - | *proline rich 3* |  | 28218614..28223801, com |
| *ABCF1* |  | - | *ATP-binding cassette, sub-family F (GCN20), member 1* |  | 28198593..28211745, com |
| *PPP1R10* |  |  | *protein phosphatase 1, regulatory subunit 10* |  | 28155859..28193657 |
| *MRPS18B* |  |  | *mitochondrial ribosomal protein S18B* |  | 28169921..28176935, com |
| *ATAT1* |  |  | *alpha tubulin acetyltransferase 1* |  | 28156562..28169425, com |
| *C23H6orf136* |  |  | *chromosome 23 open reading frame, human C6orf136* |  | 28152659..28156380, com |
| *DHX16* |  |  | *DEAH (Asp-Glu-Ala-His) box polypeptide 16* |  | 28140238..28152495 |
| *PPP1R18* |  |  | *protein phosphatase 1, regulatory subunit 18* |  | 28128194..28137023 |
| *MDC1* |  |  | *mediator of DNA-damage checkpoint 1* |  | 28116521..28128504, com |
| *NRM* |  |  | *nurim (nuclear envelope membrane protein)* |  | 28111673..28116241, com |
| *MDC1-like* | LOC100336520 |  | *mediator of DNA damage checkpoint protein 1-like* |  | 28107259..28111078 |
| *TUBB* |  |  | *tubulin, beta class I* |  | 28100876..28105330, com |
| *FLOT1* |  |  | *flotillin 1* |  | 28090029..28100348 |
| *IER3* |  |  | *immediate early response 3* |  | 28088455..28089726 |
| *LOC101905722* |  |  | *uncharacterized LOC101905722* | ncRNA | 28019005..28036808, com |
| *DDR1* |  |  | *discoidin domain receptor tyrosine kinase 1* |  | 27975618..27989666, com |
| *GTF2H4* |  |  | *general transcription factor IIH, polypeptide 4, 52kDa* |  | 27962143..27968734, com |
| *VARS2* |  |  | *valyl-tRNA synthetase 2, mitochondrial (putative)* |  | 27949619..27962028, com |
| *SFTA2* |  |  | *surfactant associated 2* |  | 27945517..27946341 |
| *DPCR1* |  |  | *diffuse panbronchiolitis critical region 1* |  | 27927103..27936148, com |
| *MUC21-like* | LOC788774 |  | *mucin-21-like* |  | 27907992..27909549, com |
| *SAP30L-like* | LOC787188 |  | *histone deacetylase complex subunit SAP30L-like* |  | 27900050..27900731, com |
| *BOLA-NC1* |  |  | *non-classical MHC class I antigen* |  | 27862921..27867793 |
| *BOLA-like* | LOC616942 |  | *major histocompatibility complex, class I, A-like* |  | 27842188..27846500 |
| *RRAGC-like* | LOC100141101 |  | *ras-related GTP-binding protein C-like* |  | 27835648..27837621, com |
| *C23H6orf15* |  |  | *chromosome 23 open reading frame, human C6orf15* |  | 27816299..27817596 |
| *CDSN* |  |  | *corneodesmosin* |  | 27808496..27812714 |
| *PSORS1C2* |  |  | *psoriasis susceptibility 1 candidate 2* |  | 27793273..27794714 |
| *CCHCR1* |  |  | *coiled-coil alpha-helical rod protein 1* |  | 27779766..27791244 |
| *TCF19* |  |  | *transcription factor 19* |  | 27775672..27779507, com |
| *POU5F1* |  |  | *POU class 5 homeobox 1* |  | 27769893..27774392 |
| *MIC1* |  |  | *major histocompatibility class I related protein* |  | 27659363..27729791 , com |
| *BOLA-like* | LOC788634 |  | *BOLA class I histocompatibility antigen, alpha chain BL3-7-like* | pseudo | 27715005..27717717 |
| *BOLA** |  |  | *MHC class I heavy chain* |  | 27679384..27692448 |
| *BOLA-A* |  |  | *major histocompatibility complex, class I, A* |  | 27646983..27692539 |
| *MIC2** |  |  | *CD99 molecule* |  | 27659409..27666210, com |
| *IFITM3-like* | LOC520748 |  | *interferon induced transmembrane protein 3 pseudogene* | pseudo | 27646134..27646583 |
| *UCR2-like* | LOC783502 |  | *cytochrome b-c1 complex subunit 2, mitochondrial pseudogene* | pseudo | 27613894..27615466 |
| *MGC126945* |  |  | *uncharacterized protein MGC126945* |  | 27600073..27604060 |
| *MCCD1* |  |  | *mitochondrial coiled-coil domain 1* |  | 27576730..27580343, com |
| *DDX39B* |  |  | *DEAD (Asp-Glu-Ala-Asp) box polypeptide 39B (Bat1)* |  | 27565924..27576721 |

*not on current assembly
