# Supplementary material for: A comprehensive mapping of the structure and gene organisation in the sheep MHC class I region
Source: BMC Genomics. 2015 Oct 19;16:810. doi: 10.1186/s12864-015-1992-4 (PMC4613773; doi:10.1186/s12864-015-1992-4)
Supplement: Additional file 4: — Amino acid sequence alignments of MHC class I genes. Description of data: Additional file 4 shows amino acid sequence alignments of MHC class I genes from selected BAC sequences published by Gao et al., 2010 and the primary sheep reference assembly for chromosome 20 (NC_019477.1) in comparison with six class I reference sequences from the IPD database. (DOCX 491 kb) [file 12864_2015_1992_MOESM4_ESM.docx]

Supplementary Figure 4. Shows an alignment of MHC class I amino acid sequences predicted from selected BAC sequences published by Gao et al, 2010 in comparison with six class I reference sequences from the IPD database (N*xxxxx) and an alternative transcript sequence AAA31566. Nomenclature for the predicted genes derived as follows: NCBI accession number, plus ‘rc’ if the sequence was reverse complemented before analysis, plus the class I gene identifier within the BAC. IPD sequences identified according to IPD allele name in the IPD-MHC Database (<http://www.ebi.ac.uk/cgi-bin/ipd/mhc/view_nomenclature.cgi?ovar.n>). AAA31566 identified as an allele of Locus 8 (_Loc8) by Ballinghall, et al 2008. A vertical box surrounds a motif in the transmembrane region important in classification of class I sequences as classical or nonclassical in cattle and sheep. Horizontal Boxes surround the extended seventh exon of a potential alternate transcript.

Supplementary Figure 5. Shows an alignment of MHC class I amino acid sequences from genes currently annotated in the primary sheep reference assembly for chromosome 20 (NC_019477.1) in comparison with six class I reference sequences from the IPD database (N*xxxxx) and an alternative transcript sequence AAA31566. Predicted sequences identified by Gene Symbol. IPD sequences identified according to IPD allele name in the IPD-MHC Database (<http://www.ebi.ac.uk/cgi-bin/ipd/mhc/view_nomenclature.cgi?ovar.n>). AAA31566 identified as an allele of Locus 8 (_Loc8) by Ballinghall, et al 2008. A vertical box surrounds a motif in the transmembrane region important in classification of class I sequences as classical or nonclassical in cattle and sheep.
